# Supplementary material for: Long-read powered viral metagenomics in the oligotrophic Sargasso Sea
Source: Nat Commun. 2024 May 14;15:4089. doi: 10.1038/s41467-024-48300-6 (PMC11094077; doi:10.1038/s41467-024-48300-6)
Supplement: Supplementary file 1 — Supplementary Information [file 41467_2024_48300_MOESM1_ESM.pdf]

Long-read powered viral metagenomics in the oligotrophic Sargasso Sea

Joanna Warwick-Dugdale[1,2 †*], Funing Tian[3 †], Michelle L Michelsen[1], Dylan R Cronin[3,4], Karen Moore[1], Audrey Farbos[1], Lauren Chittick[3], Ashley Bell[1], Ahmed A Zayed[3,4], Holger H Buchholz[1,5], Luis M Bolanos[1], Rachel J Parsons[6,7], Michael J Allen[1], Matthew B Sullivan[3,4,8], Ben Temperton[1 *]

[1] School of Biosciences, University of Exeter, Exeter, Devon EX4 4SB, United Kingdom. *Emails: jo.warwick@gmail.com b.temperton@exeter.ac.uk

[2] Plymouth Marine Laboratory, Plymouth, Devon PL1 3DH, United Kingdom

[3] Center of Microbiome Science, Department of Microbiology, Ohio State University, Columbus, OH 43210, USA

[4] EMERGE Biology Integration Institute, Ohio State University, Columbus, OH 43210, USA

[5] Department of Microbiology, Oregon State University, Corvallis, OR 97331, USA

[6] Bermuda Institute of Ocean Sciences, St.George's GE 01, Bermuda

[7] School of Ocean Futures, Arizona State University, Tempe, AZ, United States

[8] Department of Civil, Environmental, and Geodetic Engineering, Ohio State University, Columbus, OH 43210, USA

*Corresponding authors.

†These authors contributed equally: Joanna Warwick-Dugdale; Funing Tian

SUPPLEMENTARY INFORMATION

Supplementary Figures

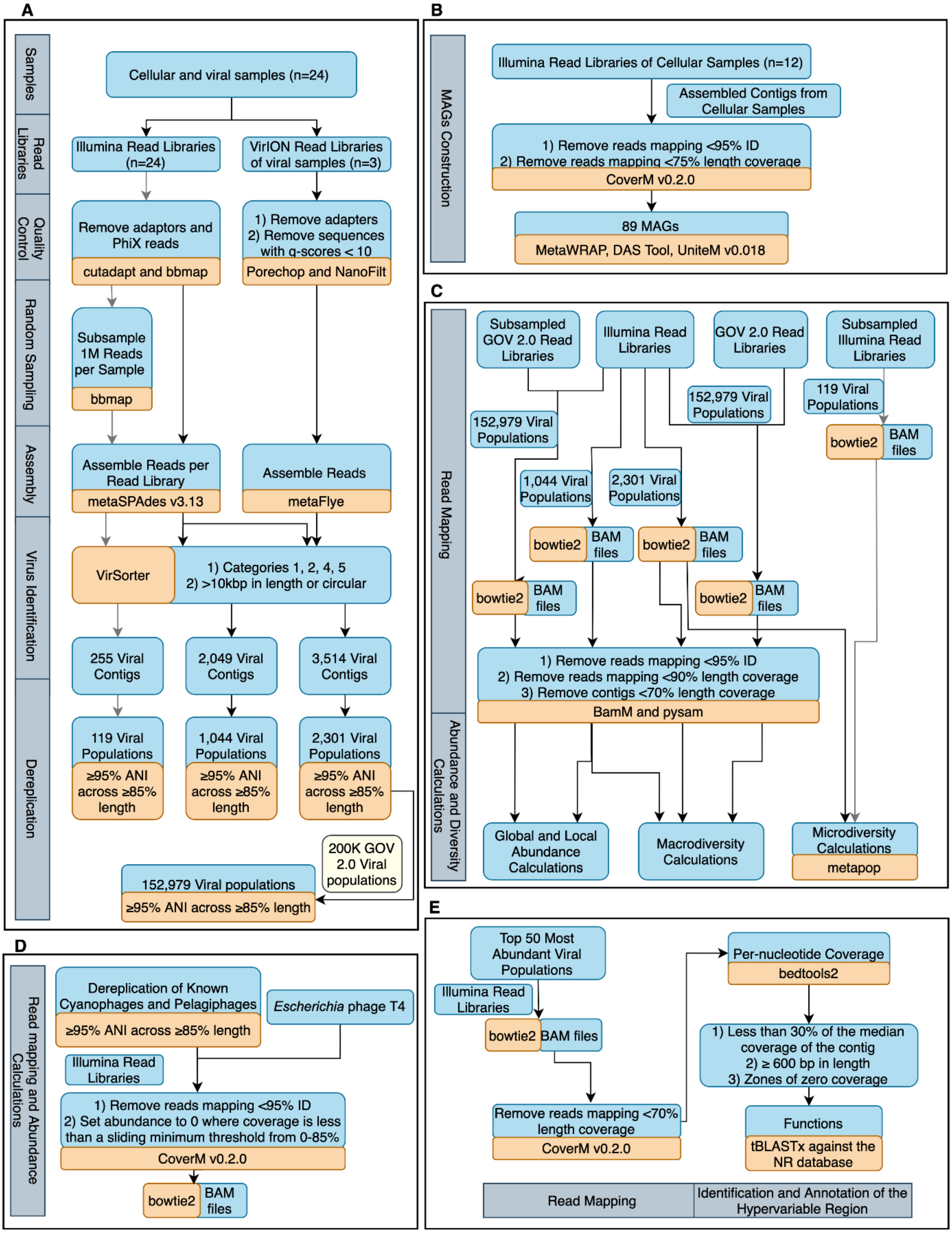

Supplementary Figure 1. Schematic representation of the bioinformatic workflow for: **A.** The assembly and identification of viral populations (VirION: Long-read

assembly and hybrid long- and short-read assembly; ANI: Average Nucleotide Identity; GOV/GOV 2.0: Global Ocean Virome 2[1]); **B.** The construction of Metagenome Assembled Genomes (MAGs); **C.** The abundance and diversity calculation of viral populations; **D.** The abundance calculation of known cyanophages and pelagiphages; **E.** Viral Hypervariable Region (HVR) recovery and identification.

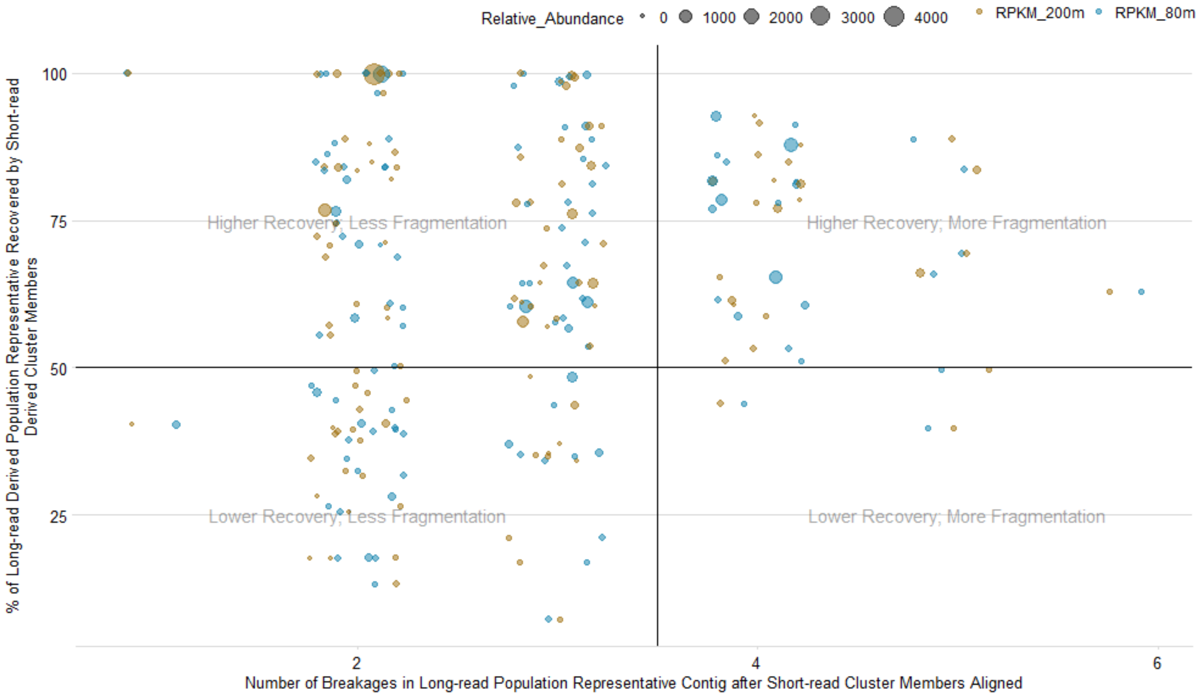
Relative_Abundance
0
1000
2000
3000
4000
RPKM_200m
RPKM_80m
% of Long-read Derived Population Representative Recovered by Short-read Derived Cluster Members
100
75
50
25
Higher Recovery, Less Fragmentation
Higher Recovery, More Fragmentation
Lower Recovery, Less Fragmentation
Lower Recovery, More Fragmentation
2
4
6
Number of Breakages in Long-read Population Representative Contig after Short-read Cluster Members Aligned

A

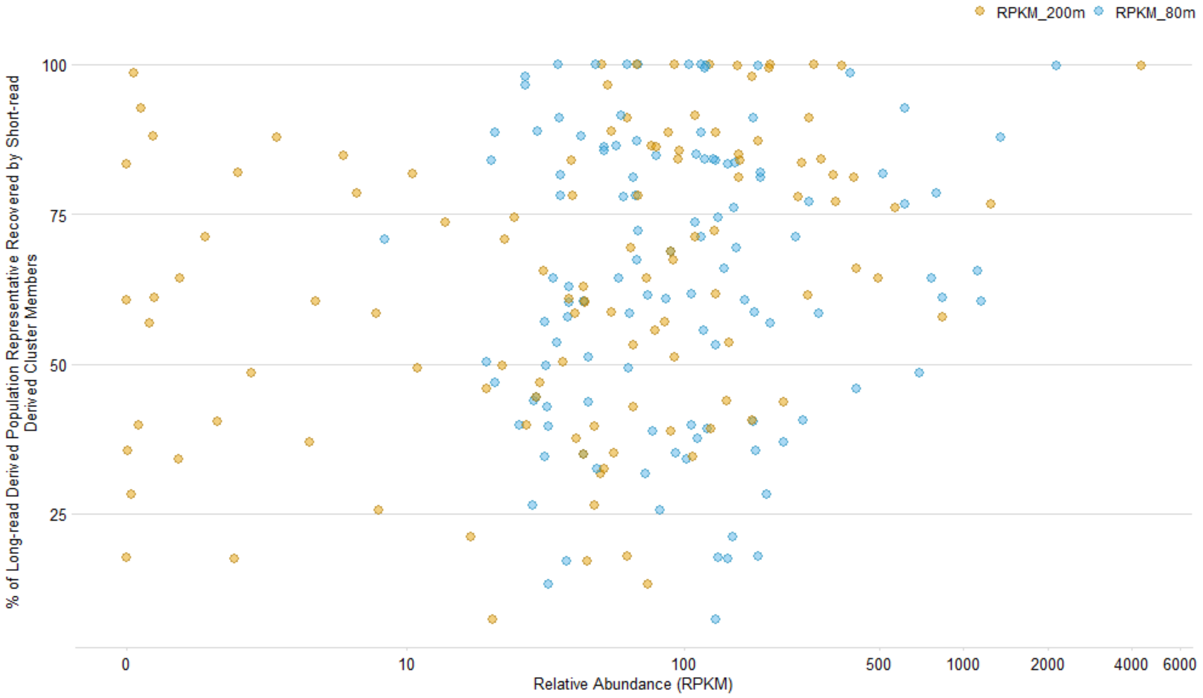
RPKM_200m
RPKM_80m
% of Long-read Derived Population Representative Recovered by Short-read Derived Cluster Members
100
75
50
25
0
10
100
500
1000
2000
4000
6000
Relative Abundance (RPKM)

B

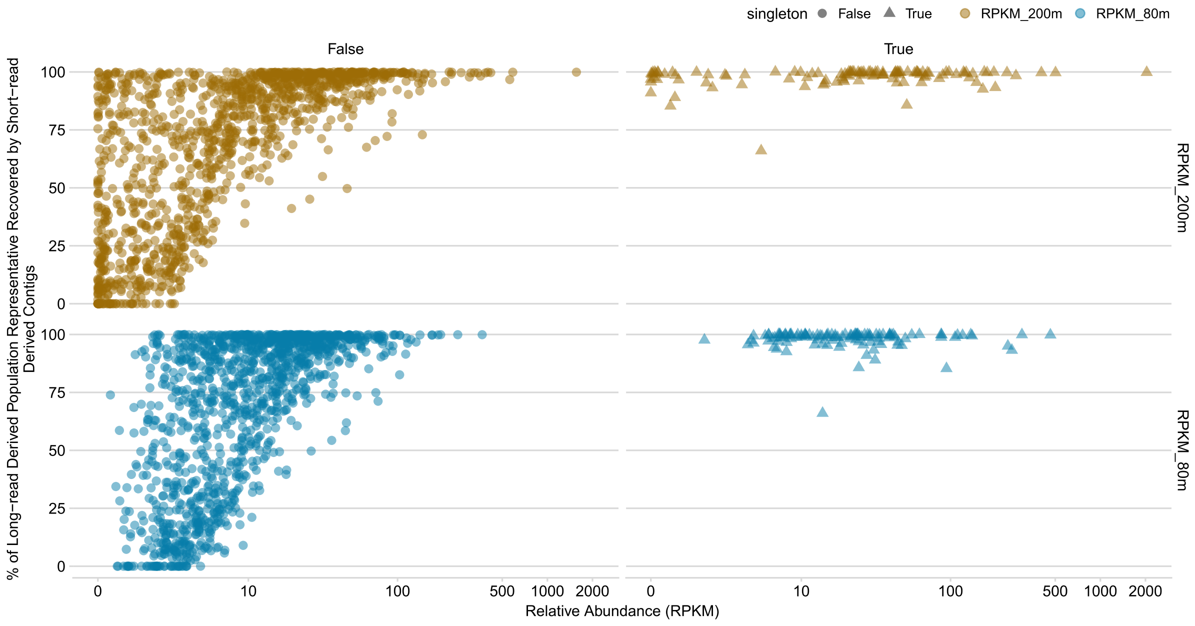

C

Supplementary Figure 2. Recovery of viral populations by long read sequencing which are missed by short reads is not a function of sequencing depth. A. Fragmentation (number of breakages) verses recovery (percentage aligned) of (*n* = 115) viral population genomes represented by long-read assemblies after alignment of short-read derived population member contigs; data-point sizes represent relative abundances (RPKM). **B.** Relative abundance (RPKM) of long-read viral population representatives that have short-read viral population members (*n* = 115), versus the percentage of long-read population representative genome recovered after alignment with short read population cluster members. **C.** Relative abundance (RPKM) of all long-read population representatives (*n* = 1410), versus the percentage of long-read population representative genome recovered after alignment with all short-read contigs >1kb.

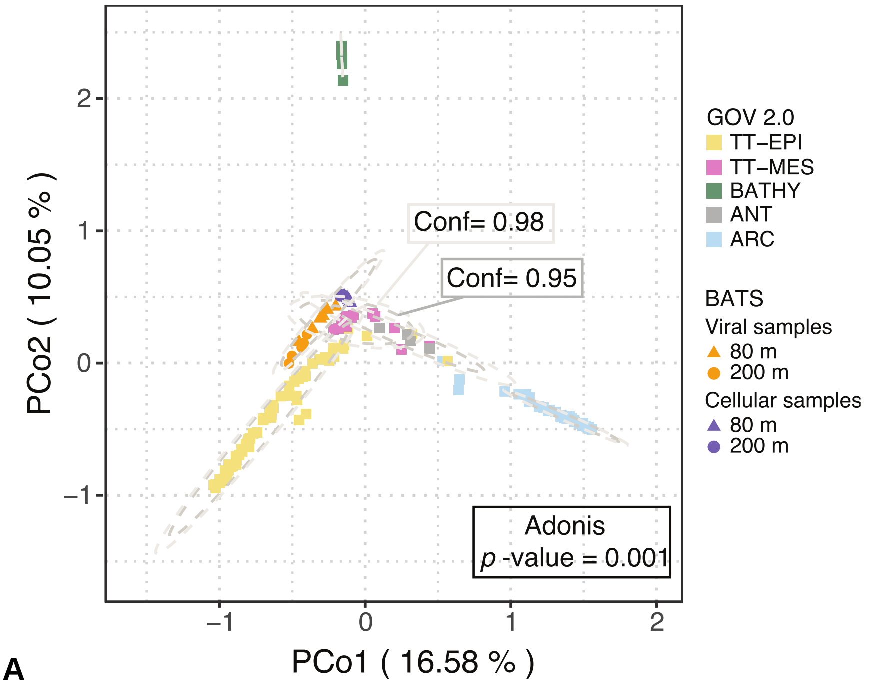

A

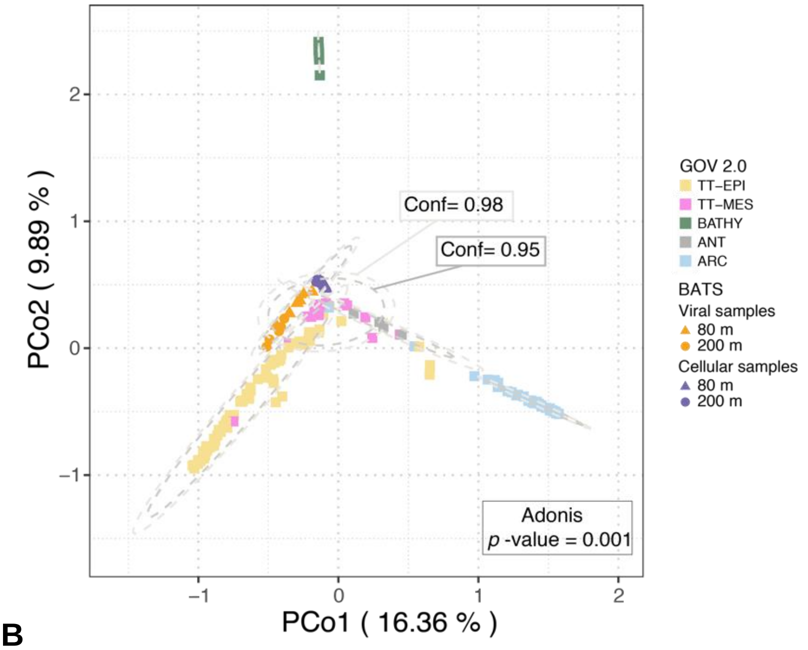

B

Supplementary Figure 3. Principal coordinates analysis (PCoA) of a Bray-Curtis dissimilarity matrix calculated from mapping Global Ocean Virome 2[1] (GOV 2.0) reads and Sargasso Sea short reads to a combined dataset of Sargasso Sea and GOV 2.0 viral populations with lengths greater than 10 kbp (*n* = 152,979; ARC: Arctic; ANT: Antarctic; TT-EPI: temperate and tropical epipelagic; TT-MES: temperate and tropical mesopelagic). **A.** Viral community structure was suggested by

ellipses drawn at 95% (inner) and 98% (outer) confidence intervals (Conf) and analysis of variance (two-sided Adonis, *p*-value = 0.001): **B.** Three outlier GOV 2.0 viromes (station 155_SUR, station 72_MES, station102_MES[1]) were not removed.

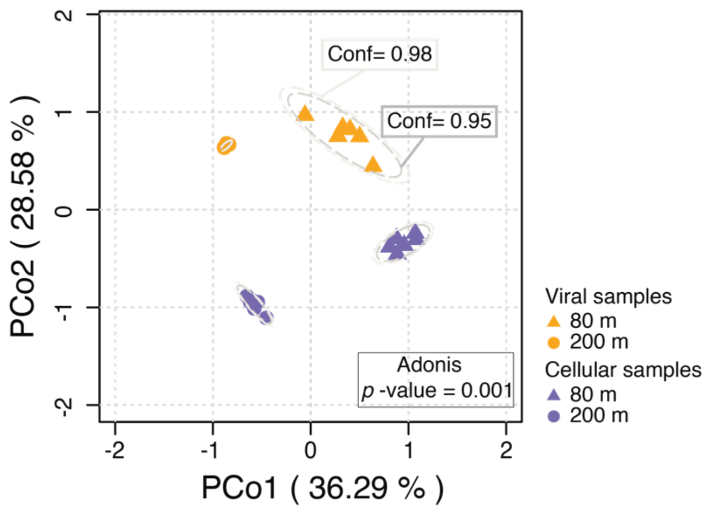

Supplementary Figure 4. Principal coordinates analysis (PCoA) of a Bray-Curtis dissimilarity matrix calculated from mapping Sargasso Sea short reads to Sargasso Sea viral populations (*n* = 1,044) derived from short-read assemblies. Viral community structure was suggested by ellipses drawn at 95% (inner) and 98% (outer) confidence (Conf) intervals and analysis of variance (two-sided Adonis, *p*-value = 0.001).

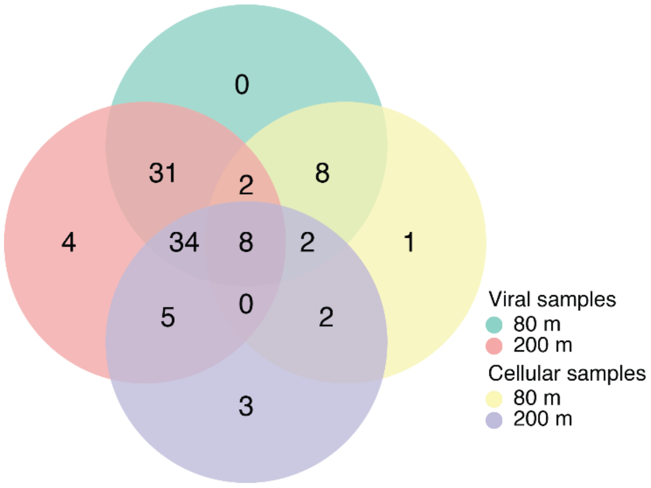

Supplementary Figure 5. Top 100 most abundant Sargasso Sea viral populations identified from (*n* = 1044) viral populations derived from short-read only assemblies: presence-absence in 80 m viral samples, 80 m cellular samples, 200 m viral samples, and 200 m cellular samples.

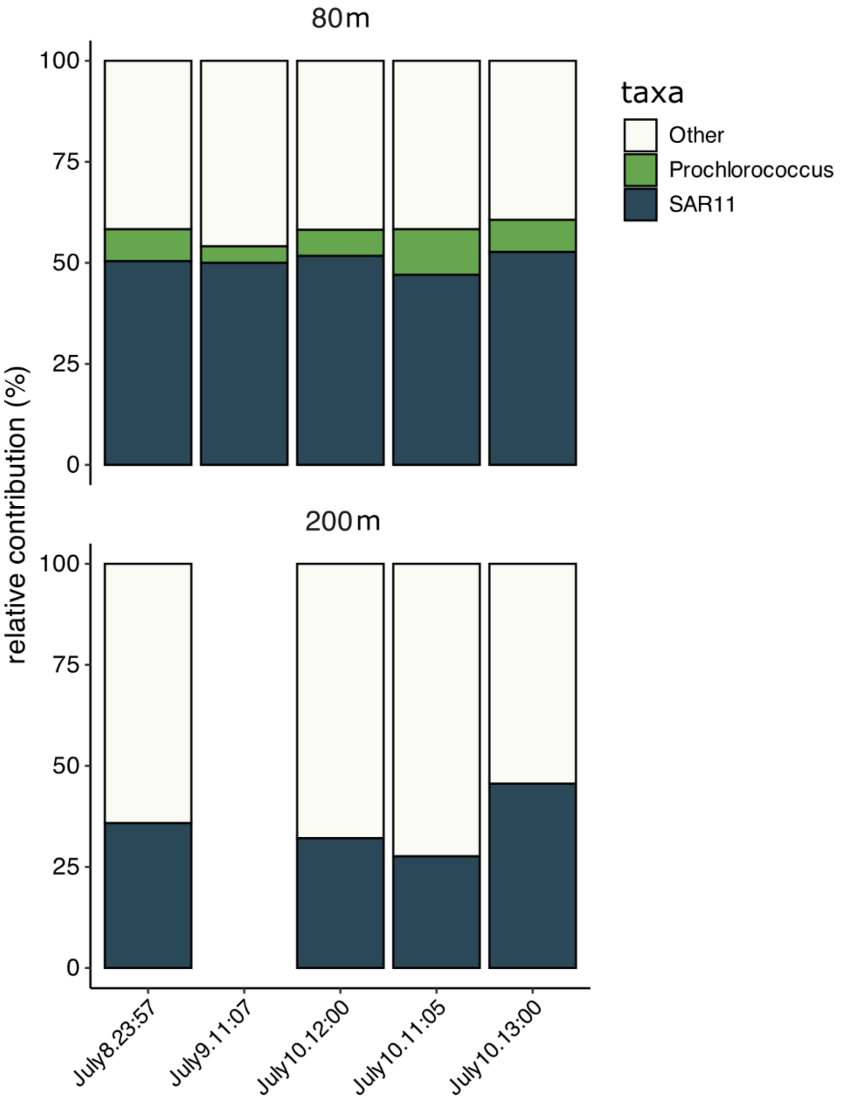

Supplementary Figure 6. Relative abundance of the SAR11 (coloured navy) and *Prochlorococcus* (coloured green) Amplicon Sequence Variants (ASVs) contribution to the total amplicons sequenced during the sampling in cruise. Top panel depicts the relative abundances at a depth of 80 m and the bottom panel at 200 m.

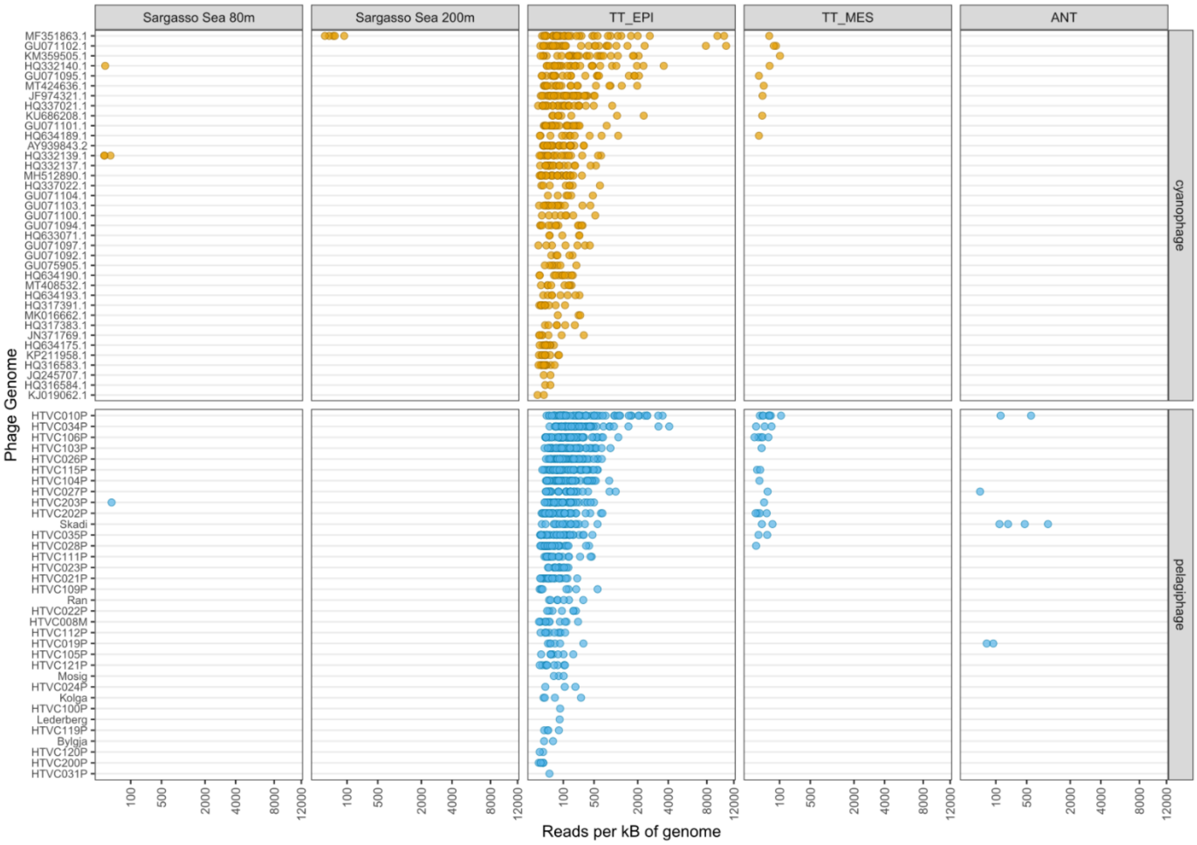
Sargasso Sea 80m
Sargasso Sea 200m
TT_EPI
TT_MES
ANT
cyanophage
pelagiphage
Phage Genome
Reads per kB of genome
MF351863.1
GU071102.1
KM359505.1
HQ332140.1
GU071095.1
MT424636.1
JF974321.1
HQ337021.1
KU686208.1
GU071101.1
HQ634189.1
AY939843.2
HQ332139.1
HQ332137.1
MH512890.1
HQ337022.1
GU071104.1
GU071103.1
GU071100.1
GU071094.1
HQ633071.1
GU071097.1
GU071092.1
GU075905.1
HQ634190.1
MT408532.1
HQ634193.1
HQ317391.1
MK016662.1
HQ317383.1
JN371769.1
HQ634175.1
KP211958.1
HQ316583.1
JQ245707.1
HQ316584.1
KJ019062.1
HTVC010P
HTVC034P
HTVC106P
HTVC103P
HTVC026P
HTVC115P
HTVC104P
HTVC027P
HTVC203P
HTVC202P
Skadi
HTVC035P
HTVC028P
HTVC111P
HTVC023P
HTVC021P
HTVC109P
Ran
HTVC022P
HTVC008M
HTVC112P
HTVC019P
HTVC105P
HTVC121P
Mosig
HTVC024P
Kolga
HTVC100P
Lederberg
HTVC119P
Bylgja
HTVC120P
HTVC200P
HTVC031P
100
500
2000
4000
8000
12000

A

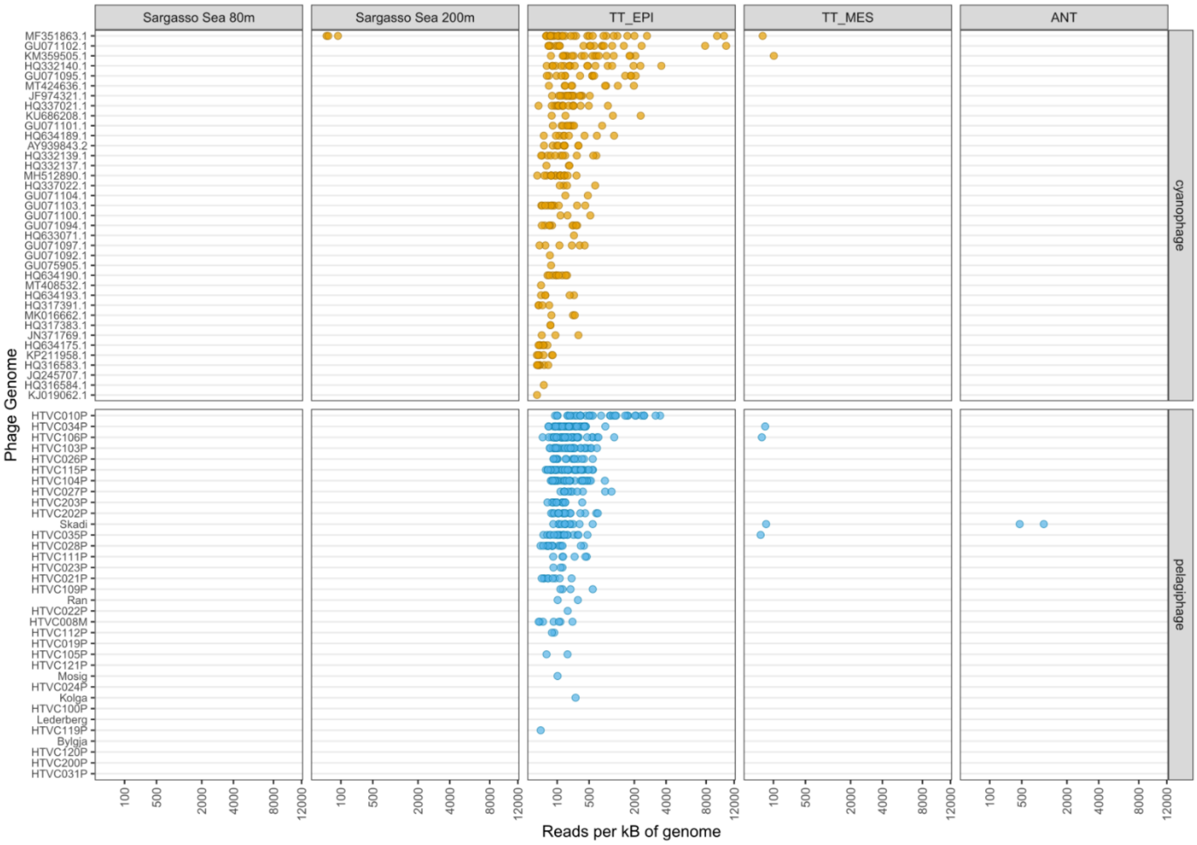
Sargasso Sea 80m
Sargasso Sea 200m
TT_EPI
TT_MES
ANT
cyanophage
pelagiphage
Phage Genome
MF351863.1
GU071102.1
KM359505.1
HQ332140.1
GU071095.1
MT424636.1
JF974321.1
HQ337021.1
KU686208.1
GU071101.1
HQ634189.1
AY939843.2
HQ332139.1
HQ332137.1
MH512890.1
HQ337022.1
GU071104.1
GU071103.1
GU071100.1
GU071094.1
HQ633071.1
GU071097.1
GU071092.1
GU075905.1
HQ634190.1
MT408532.1
HQ634193.1
HQ317391.1
MK016662.1
HQ317383.1
JN371769.1
HQ634175.1
KP211958.1
HQ316583.1
JQ245707.1
HQ316584.1
KJ019062.1
HTVC010P
HTVC034P
HTVC106P
HTVC103P
HTVC026P
HTVC115P
HTVC104P
HTVC027P
HTVC203P
HTVC202P
Skadi
HTVC035P
HTVC028P
HTVC111P
HTVC023P
HTVC021P
HTVC109P
Ran
HTVC022P
HTVC008M
HTVC112P
HTVC019P
HTVC105P
HTVC121P
Mosig
HTVC024P
Kolga
HTVC100P
Lederberg
HTVC119P
Bylgja
HTVC120P
HTVC200P
HTVC031P
100
500
2000
4000
8000
12000
Reads per kB of genome

B

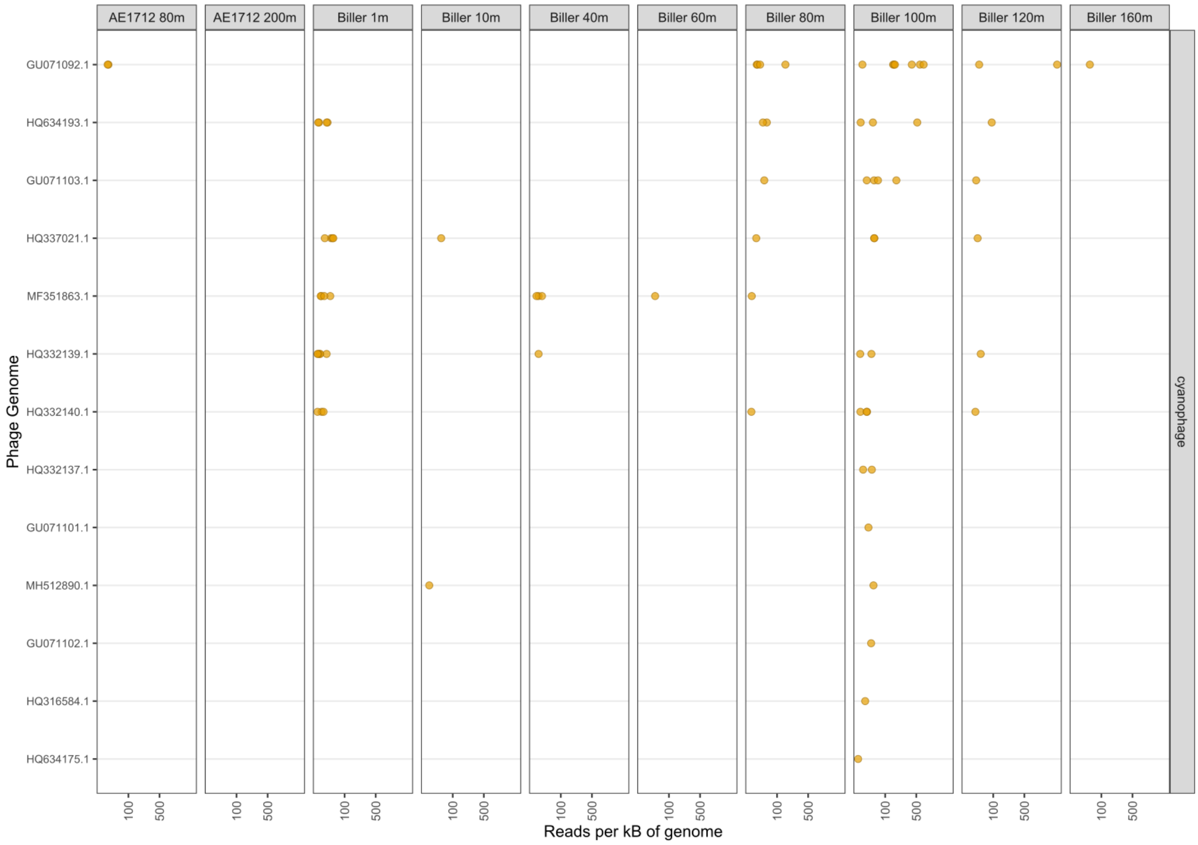
AE1712 80m
AE1712 200m
Biller 1m
Biller 10m
Biller 40m
Biller 60m
Biller 80m
Biller 100m
Biller 120m
Biller 160m
cyanophage
Phage Genome
GU071092.1
HQ634193.1
GU071103.1
HQ337021.1
MF351863.1
HQ332139.1
HQ332140.1
HQ332137.1
GU071101.1
MH512890.1
GU071102.1
HQ316584.1
HQ634175.1
100
500
Reads per kB of genome

C

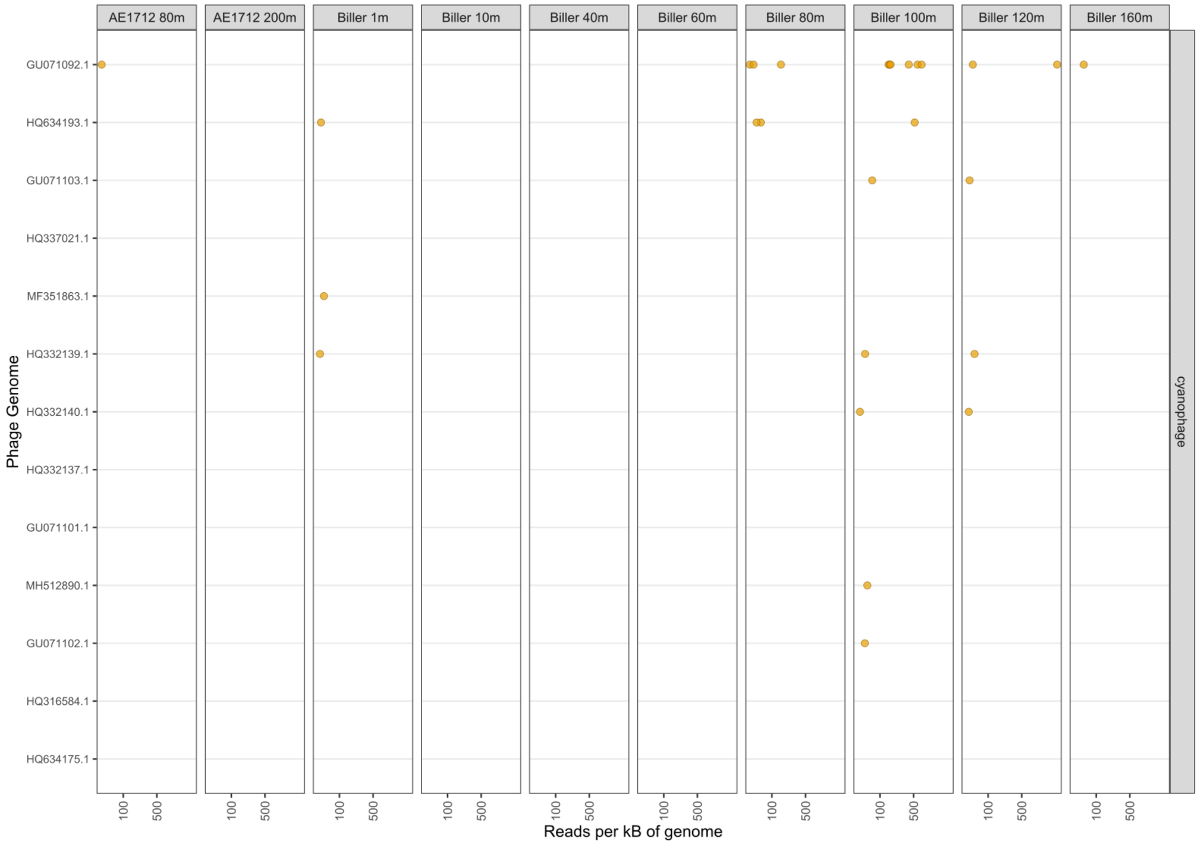

D

Supplementary Figure 7. Abundance (RPKM) of known cyanophages (coloured yellow) and pelagiphages (coloured blue) in short-read data from: Viral fraction samples from this study and Global Ocean Virome 2.0[1] samples (ANT: Antarctic; TT-EPI: temperate and tropical epipelagic; TT-MES) at minimum genome coverage cut-off values of: **A.** 40%; **B.** 80%. Cellular fraction samples from this study and published cellular metagenome samples from the Sargasso Sea[2] (Biller) at minimum genome coverage cut-off values of: **C.** 40%; **D.** 70%**.** Note that no known pelagiphages were detected at ≥40% minimum genome coverage cut-off in any Sargasso Sea cellular fraction metagenomes. Cleaned Illumina sequences were competitively recruited (at ≥90% read length at ≥95% identity) to a dereplicated (at ≥ 95% nucleotide identity across ≥ 85% genome length) database of all published cyanophage and pelagiphage isolate genomes (plus control *Escherichia* phage T4).

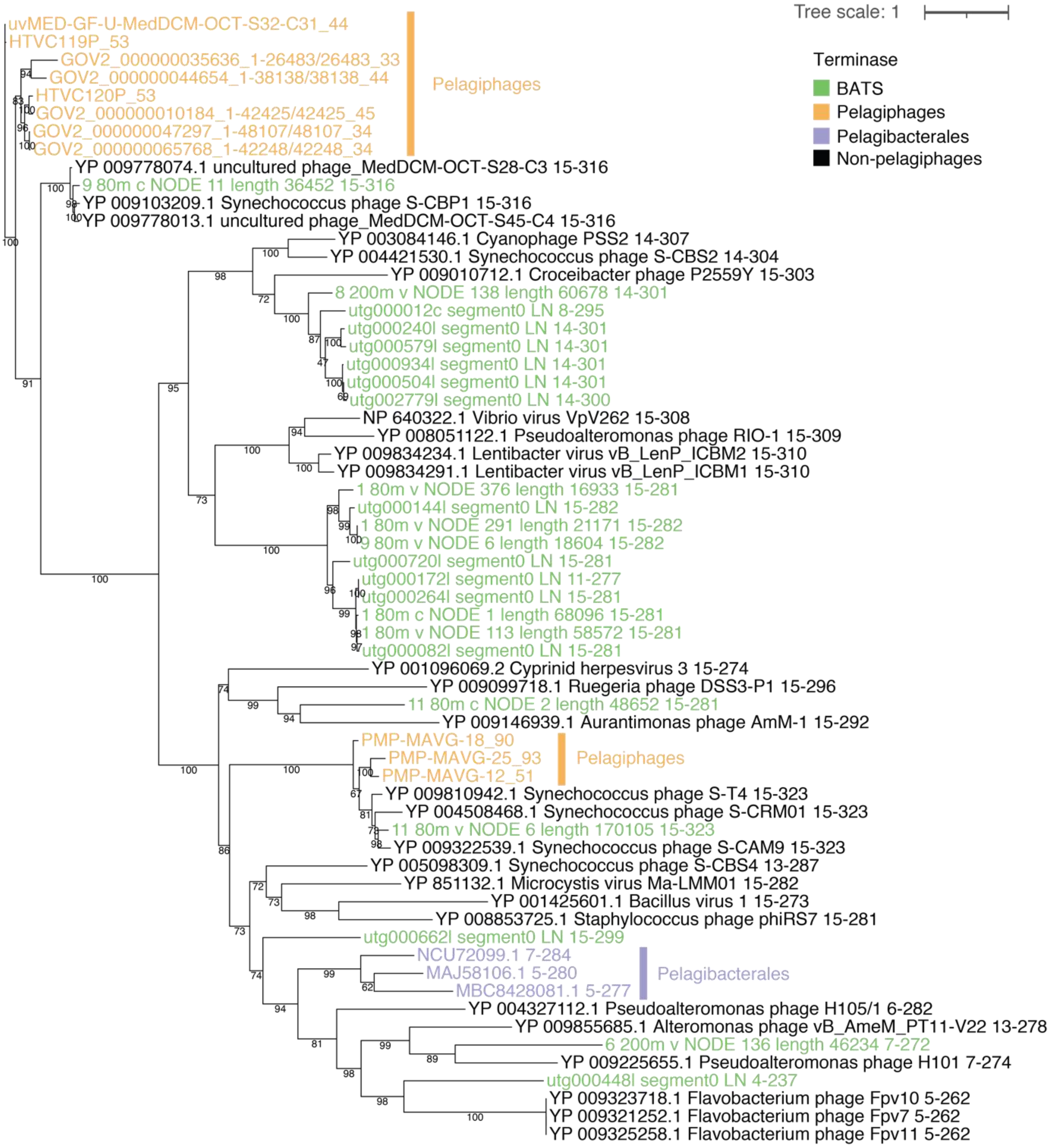

Supplementary Figure 8. Phylogenetic tree constructed using terminase (*TerL*) genes from published pelagiphage genomes (orange), non-pelagiphages (black), *Pelagibacterales* (purple; identified via BLAST hits against terminase from known pelagiphages) and the 100 most abundant Sargasso Sea viral populations (green; *TerL* genes aligned using E-INS-i strategy; bootstrapping: 1000 iterations; clades subsampled for clarity).

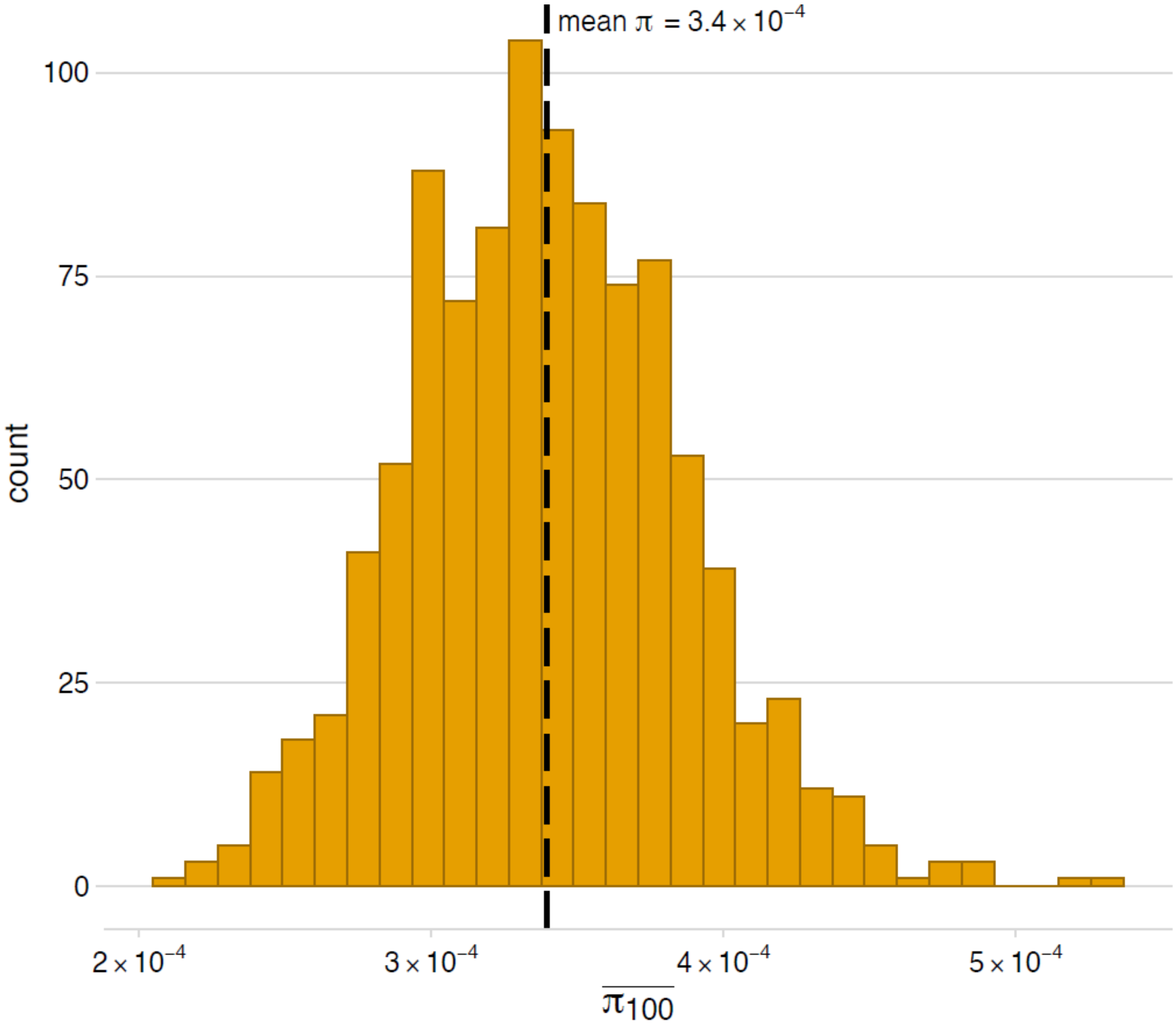

Supplementary Figure 9. Average microdiversity (π) across Sargasso Sea viruses from both depths sampled (80 m and 200 m). Mean π was calculated as Gregory *et al* [1]: 100 π values were randomly subsampled from short-read Sargasso Sea viromes (*n* = 2,049) with replacement; the distribution mean π was generated via bootstrapping (1000 iterations), and 95% confidence intervals were calculated using the quantiles of this distribution.

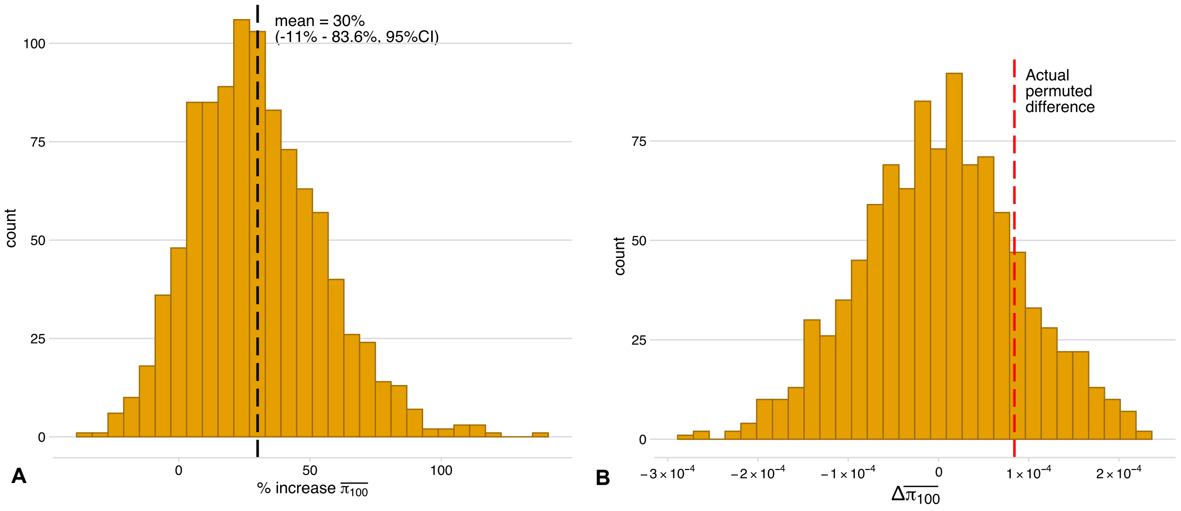

Supplementary Figure 10. The permuted percentage increase of ~30% between average microdiversity values (mean π; calculated as Gregory *et al*[1]) of Sargasso Sea viruses from 80 m and 200 m (respectively): **A.** 30.041% (-11.381 - 83.604, 95% CI), was not found to be significant; **B.** permuted significance test: p = 0.164; under the null model, a difference in the two populations at least as extreme as the actual measured difference (red line) was observed 164 times out of 1000 permutations).

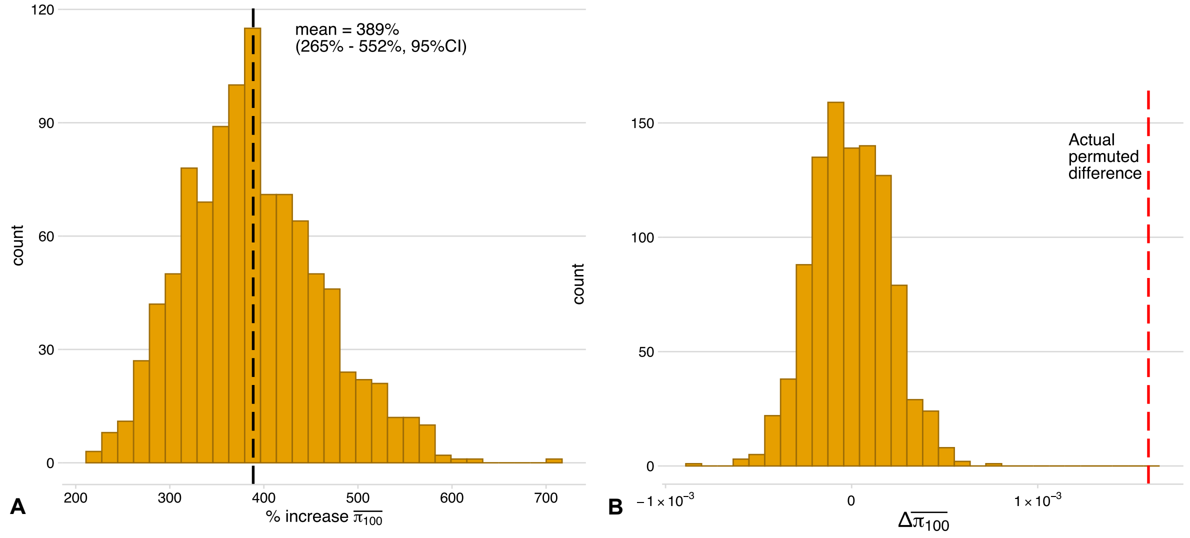

Supplementary Figure 11. The permuted percentage increase of ~390% between average microdiversity values (mean π; calculated as Gregory *et al*[1]) of Sargasso Sea viruses captured by VirION (n = 1,465) compared to those assembled from short

reads (*n* = 2,049): **A.** 388.668% (264.559 - 551.95%, 95% CI), was highly significant; **B.** permuted significance test: *p* <0.001; under the null model, a difference in the two populations at least as extreme as the actual measured difference (red line) was not observed (0 times out of 1000 permutations).

Supplementary Tables

Contig code	HVR length	Protein name/s	Source/s
utg000082	993	virion structural protein	*Synechococcus* phage S-CAM1 isolates 0910CC29; 0810SB17; 0809CC03; 0310NB17
utg000124	849	gp34; conserved hypothetical protein; **class I ribonucleotide reductase beta subunit; class I ribonucleotide reductase alpha subunit; ribonucleotide reductase domain-containing protein**; hypothetical protein ('similar to gp34'); hypothetical protein P60_gp20; hypothetical protein P60_gp21; **MazG nucleotide pyrophosphohydrolase**;	Cyanophages NATL1A-7, NATL2A-133, 9515-10a; *Synechococcus* phages S-CBP1, S-CBP3; *Prochlorococcus* phages P-SSP6, P-SSP7; Uncultured marine virus isolate Oxic3_4
utg000124	1122	**T7-like tail fiber** ("gp17"); major capsid protein; **phage tail fiber-like protein**	*Prochlorococcus* phages P-SSP7, P-SSM2; *Synechococcus* phage S-CBP1
utg000124	1760	**HNH endonuclease:HNH nuclease;** gp50; **HNH nuclease**; putative endonuclease; **phage tail fiber-like protein**; possible endonuclease; **HNH endonuclease**; HNH endonuclease family protein	*Prochlorococcus* phages P-SSP6, P-SSP7; P-SSP10, P-SSM2; Cyanophage 9515-10a; *Prochlorococcus* sp. RS04, MIT 0604; *Prochlorococcus marinus* str. MIT 9215;
utg000504	1986	Hypothetical protein	**Uncultured virus clone vSAG-88-3-L14**
utg000504	3406	**terminase small subunit; exonuclease; putative exonuclease**; Hypothetical protein	Pelagibacter phage HTVC027P; Uncultured virus clone vSAG-41-H17; Pelagibacter phage HTVC023P
utg001170	933	hypothetical protein ('**putative SAR11 Pelagibacter phage**')	Uncultured Mediterranean phage uvMED

Supplementary Table 1. Predicted function of candidate hypervariable genomic regions (HVRs) in Sargasso Sea viruses obtained with the VirION pipeline. Illumina

reads were mapped to the top 50 most abundant Sargasso Sea viral population representatives (at 95% identity and 70% coverage), and the encoded function of putative HVR regions that were ≥600 bp long and contained areas of zero coverage were investigated (via a tBLASTx search against the NCBI NR database).

Statistic / Sample code	BS1	BS3	BS5	80m_unassigned
Mean read length (bp)	4,100.1	3,840.1	3,738.8	3,927.9
Mean read quality	12.3	12.6	12.6	12.0
Median read length (bp)	3,662.0	3,534.0	3,441.0	3,583.0
Median read quality	12.6	12.8	12.8	12.3
Number of reads	363,245	481,694	509,715	2,399,558
Read length N50 (bp)	5,042.0	4,370.0	4,285.0	4,588.0
Total bases (Mbp)	1489.3	1849.7	1905.7	9425.2
% reads >Q10	88.6	89.7	90.1	82.8
Longest read >Q10 (bp)	27114	20909	24884	32222

Supplementary Table 2. Summary statistics for MinION long-read sequencing of Sargasso Sea viral communities (calculated with NanoStat[3]). Barcoded libraries for three samples taken from 80 m depth (encoded: BS1; BS3; BS5) were sequenced. Barcode ligation failed in >42% of reads; the sequencing depth remaining per sample was too low for successful downstream processing, so for further analysis sequences were pooled.

Supplementary Discussion

Hypervariable regions of Sargasso Sea Viral genomes encode proteins putatively associated with host recognition, DNA synthesis and packaging

Bacterioplankton communities are the primary drivers of the global carbon cycle[4], and studies of community composition in the Sargasso Sea have provided valuable insights towards understanding productivity and nutrient cycling on the global scale[5,6]. Sargasso Sea microbial communities are distinct across seasonal and depth gradients[7]. Depth-specific niches are occupied by ecologically important heterotrophs, such as Pelagibacter (SAR11), Chloroflexi (SAR202), Deltaproteobacteria (SAR324) and Marinomicrobia (SAR406)[8–11], and the

phototrophs *Prochlorococcus*[12] and *Synechococcus*[13]. *Prochlorococcus* and SAR11 (Pelagibacter) are the dominant phototrophs and heterotrophs in epipelagic surface waters, respectively[7,14]. The extremely low levels of nutrients in the Sargasso Sea photic zone have constrained the diversity of the microbial communities, as revealed by 16S rRNA community profiling[7]. However, metagenomic surveys revealed fine-scale, intraspecific diversity of *Prochlorococcus* and SAR11[6], which were later assessed using population genomics as comprising of co-existing populations[15–17]. Through time it has become clear that these populations have large shared or 'core-' genomic regions that are chequered with 'flexible' genes/regions, as well as 'hypervariable regions' (HVRs) or genomic islands[18] where most niche-differentiation is thought to occur for these taxa[15–17,19–21]. HVRs are identified when short reads from metagenomic datasets are mapped against genomes from the same environment to reveal regions of very low read recruitment indicating a putative HVR[18,22].

Like their prokaryotic hosts, phage genomes include both conserved regions, and hypervariable regions (HVRs), which vary highly between otherwise closely related viruses[23,24]. Virally-encoded hypervariable regions can encode for host recognition structures and tail proteins[23,25], as well as putative internal virion proteins, terminases and ribonucleases[24,26]. Previous work has shown that the hybrid, VirION approach improves capture of niche-defining HVRs[26]. Our search for Sargasso Sea viral HVRs was rigorously constrained to only include regions with a minimum size ≥600 bp with near zero coverage in the top 50 most abundant viral populations to avoid false positive detection of HVRs from low-coverage taxa. Application of these parameters revealed nine putative HVRs encoded in five viral contigs; alignment of these to the NCBI NR database via a tBLASTx search (Supplementary Table S1) produced functional annotations for seven of the HVRs encoded in four contigs.

One advantage of long-read sequencing is the ability to identify otherwise missed HVRs – areas of viral genomes likely associated with host-virus interaction and 'arms race' adaptations (as proposed in host genomes[27]). Two viral contigs encoded four HVRs containing putative ORFs with homology to proteins from *Synechococcus* phages, *Prochlorococcus* phages and cyanophages, and

comprised: structural proteins including T7-like tail fibre, tail fibre-like proteins, capsid proteins; MazG nucleotide pyrophosphohydrolase; ribonucleotide reductases; HNH nucleases/endonucleases (part of a homing endonuclease domain characterised by histidine and asparagine residues); and many hypothetical proteins (43% of annotations). T7-like tail fibre proteins previously recovered from pelagiphage HVRs were assumed to denote regions of rapid evolution associated with host recognition and a co-evolutionary arms race[24]. This evidence suggests that phages of phototrophic hosts may also carry such genes within HVRs, supporting the paradigm of viral genome hypervariable regions as hotspots in the evolution of viral-host interactions[28]. Many HVR annotations noted here seemed tentatively unconnected to host recognition. MazG nucleotide pyrophosphohydrolase may function in phage DNA synthesis. A previously hypothesised role in phage mediated regulation of the stringent response (i.e., host reaction to nutrient deprivation[29]) has recently been revised, and MazG is now thought to enable recycling of host DNA via the hydrolysis of deoxyribonucleotides[30]. Enzymes such as ribonucleotide reductase (RNR), and HNH nucleases and endonucleases are predicted to function in phage DNA synthesis and packaging (respectively). RNR is a viral AMG that catalyses the formation of deoxyribonucleotides from ribonucleotides for the production of progeny DNA[31,32] . RNR genes are abundant and under selective evolutionary pressure in environmental viral assemblages[33]. HNH proteins function in conjunction with terminase in DNA cleavage for phage morphogenesis and are widespread in long-tailed phages[34]. HNH nucleases have been identified in phages from disparate environments, including a deep-sea thermophilic bacteriophage[35] and ‘hidden’ prophage of a cultured marine *Roseobacter* [36].

Why genes for DNA synthesis and packaging enzymes, and even genes related to viral structure (i.e., encoding virion and capsid proteins) have been observed within HVRs may reflect an aspect of phage biology not yet understood[24]. However, the detection of HNH nucleases here could suggest that the HVRs of viruses infecting phototrophs encode proteins to counter anti-phage defences beyond adsorption and injection of viral DNA. A potential mechanism for overcoming host defence is concerned with avoidance of Restriction-Modification (RM) systems which have been identified in multiple strains of *Prochlorococcus*, but are rare in SAR11[37,38]. RM systems identify and destroy viral DNA: phages can evade detection

by the removal, under-representation or mutation of the restriction-site sequences recognised by the host cell[39,40]. Phage encoded HNH type nucleases have been identified as likely protagonists in viral genomic rearrangement[41], so have the potential to assist phage avoidance of host RM systems via this route.

The remaining three HVRs encoded by two contigs may have derived from pelagiphages. Exonuclease, terminase and hypothetical protein annotations were sourced from pelagiphages HTVC027P and HTV023P, a putative pelagiphage acquired through metagenomic fosmids (from the Mediterranean deep chlorophyll maximum[42], and viral genomes recovered from single-virus genomics, including a virus sampled from the bathypelagic ('vSAG-88-3-L14'), and another described as one of the most abundant dsDNA viruses in the surface global marine virosphere (at species level; 'vSAG-41-H17')[43]. Like HNH proteins, exonucleases and terminases are predicted to function in phage DNA packaging[34,44], and have been previously identified in pelagiphage genomes[45,46], and viral metagenomic HVRs[24,26]. However, it is worth noting that the terminase located in the HVR did not cluster with terminases from known pelagiphages in a phylogenetic tree (Figure S8) and it is unknown as to whether HVR structure provides a robust signal for host association.

Does low host macrodiversity diversity promote high viral microdiversity in the Sargasso Sea?

At species level (macrodiversity), bacterial diversity is negatively correlated to the magnitude of Net Heat Flux (NHF; i.e. air-sea flux of heat into an oceanic system)[47]. In accordance with the Royal Family Model[48], the strong positive NHF[49,50] and associated low macrodiversity in the host community[7] of the summer-stratified Sargasso Sea could constrain viral evolution to fine-scale, nucleotide level changes that maximise niche-filling, whilst increasing microdiversity, amongst the well-established and well-adapted host-communities. This putative mechanism may explain the strong positive correlation previously observed between viral microdiversity and PAR (Photosynthetic Active Radiation)[1]. This idea is corollary to the proposition that increased fine-scale viral microdiversity may decrease more coarse-level diversity by promoting competitive exclusion[51], proposed to explain regional scale diversity patterns observed at a in the GOV 2.0 dataset[1]

Supplementary References

1. Gregory, A. C. *et al.* Marine DNA Viral Macro- and Microdiversity from Pole to Pole. *Cell* **177**, 1109-1123.e14 (2019).

2. Biller, S. J. *et al.* Data descriptor: Marine microbial metagenomes sampled across space and time. *Sci Data* **5**, (2018).

3. De Coster, W., D'Hert, S., Schultz, D. T., Cruts, M. & Van Broeckhoven, C. NanoPack: Visualizing and processing long-read sequencing data. *Bioinformatics* **34**, 2666–2669 (2018).

4. Falkowski, P. G., Fenchel, T. & Delong, E. F. The Microbial Engines That Drive Earth's Biogeochemical Cycles. *Science (1979)* **320**, 1034–1039 (2008).

5. Carlson, C. A. *et al.* Seasonal dynamics of SAR11 populations in the euphotic and mesopelagic zones of the northwestern Sargasso Sea. *ISME Journal* **3**, 283–295 (2009).

6. Venter, J. C. *et al.* Environmental Genome Shotgun Sequencing of the Sargasso Sea. *Science (1979)* **304**, 66–74 (2004).

7. Treusch, A. H. *et al.* Seasonality and vertical structure of microbial communities in an ocean gyre. *ISME J* **3**, 1148–1163 (2009).

8. Giovannoni, S. J. Genetic diversity in Sargasso sea bacterioplankton. *Nature* **345**, 183–187 (1990).

9. Giovannoni, S. J., Rappé, M. S., Vergin, K. L. & Adair, N. L. 16S rRNA genes reveal stratified open ocean bacterioplankton populations related to the green non-sulfur bacteria. *Proc Natl Acad Sci U S A* **93**, 7979–7984 (1996).

10. Gordon, D. A. & Giovannoni, S. J. Detection of stratified microbial populations related to Chlorobium and Fibrobacter species in the Atlantic and Pacific Oceans. *Appl Environ Microbiol* **62**, 1171–1177 (1996).

11. Wright, T. D., Vergin, K. L., Boyd, P. W. & Giovannoni, S. J. A novel δ-subdivision proteobacterial lineage from the lower ocean surface layer. *Appl Environ Microbiol* **63**, 1441–1448 (1997).

12. Moore, L. & Rocap, G. Physiology and molecular phylogeny of coexisting Prochlorococcus ecotypes. *Nature* **576**, 220–223 (1998).

13. Lomas, M. W. *et al.* Two decades and counting: 24-years of sustained open ocean biogeochemical measurements in the Sargasso Sea. *Deep Sea Research Part II: Topical Studies in Oceanography* **93**, 16–32 (2013).

14. Wang, C. & Malanotte-Rizzoli, P. Diagnosis of physical and biological controls on phytoplankton distribution in the Sargasso Sea. *Journal of Ocean University of China* **13**, 32–44 (2014).

15. Kashtan, N. *et al.* Single-cell genomics reveals hundreds of coexisting subpopulations in wild Prochlorococcus. *Science (1979)* **344**, 416–420 (2014).

16. Wilhelm, L. J., Tripp, H. J., Givan, S. A., Smith, D. P. & Giovannoni, S. J. Natural variation in SAR11 marine bacterioplankton genomes inferred from metagenomic data. *Biol Direct* **2**, 1–19 (2007).

17. Ulloa, O. *et al.* The cyanobacterium Prochlorococcus has divergent light-harvesting antennae and may have evolved in a low-oxygen ocean. *PNAS* **118**, (2021).

18. Coleman, M. L. *et al.* Genomic islands and the ecology and evolution of Prochlorococcus. *Science (1979)* **311**, 1768–1770 (2006).

19. López-Pérez, M., Haro-Moreno, J. M., Coutinho, F. H., Martinez-Garcia, M. & Rodriguez-Valera, F. The Evolutionary Success of the Marine Bacterium SAR11 Analyzed through a Metagenomic Perspective. *mSystems* **5**, 1–13 (2020).

20. Kettler, G. C. *et al.* Patterns and implications of gene gain and loss in the evolution of Prochlorococcus. *PLoS Genet* **3**, 2515–2528 (2007).

21. Cordero, O. X. & Polz, M. F. Explaining microbial genomic diversity in light of evolutionary ecology. *Nat Rev Microbiol* **12**, 263–273 (2014).

22. Rusch, D. B. *et al.* The Sorcerer II Global Ocean Sampling expedition: Northwest Atlantic through eastern tropical Pacific. *PLoS Biol* **5**, 0398–0431 (2007).

23. Angly, F. *et al.* Genomic analysis of multiple Roseophage SIO1 strains. *Environ Microbiol* **11**, 2863–2873 (2009).

24. Mizuno, C. M., Ghai, R. & Rodriguez-Valera, F. Evidence for metaviromic islands in marine phages. *Front Microbiol* **5**, 1–10 (2014).

25. Garcia-Heredia, I. *et al.* Reconstructing viral genomes from the environment using fosmid clones: The case of haloviruses. *PLoS One* **7**, (2012).

26. Warwick-Dugdale, J. *et al.* Long-read viral metagenomics enables capture of abundant and microdiverse viral populations and their niche-defining genomic islands. *PeerJ* **7**, e6800 (2019).

27. Zhao, Y. *et al.* Abundant SAR11 viruses in the ocean. *Nature* **494**, 357–60 (2013).

28. Avrani, S., Wurtzel, O., Sharon, I., Sorek, R. & Lindell, D. Genomic island variability facilitates Prochlorococcus-virus coexistence. *Nature* **474**, 604–608 (2011).

29. Bryan, M. J. *et al.* Evidence for the intense exchange of MazG in marine cyanophages by horizontal gene transfer. *PLoS One* **3**, 1–12 (2008).

30. Rihtman, B. *et al.* Cyanophage MazG is a pyrophosphohydrolase but unable to hydrolyse magic spot nucleotides. *Environ Microbiol Rep* **11**, 448–455 (2019).

31. Nordlund, P. & Reichard, P. Ribonucleotide reductases. *Annu Rev Biochem* **75**, 681–706 (2006).

32. Thompson, L. R. *et al.* Phage auxiliary metabolic genes and the redirection of cyanobacterial host carbon metabolism. *Proceedings of the National Academy of Sciences* **108**, E757–E764 (2011).

33. Dwivedi, B., Xue, B., Lundin, D., Edwards, R. A. & Breitbart, M. A bioinformatic analysis of ribonucleotide reductase genes in phage genomes and metagenomes. *BMC Evol Biol* **13**, 1–17 (2013).

34. Kala, S. *et al.* HNH proteins are a widespread component of phage DNA packaging machines. *Proc Natl Acad Sci U S A* **111**, 6022–6027 (2014).

35. Zhang, L. *et al.* Structural and functional characterization of deep-sea thermophilic bacteriophage GVE2 HNH endonuclease. *Sci Rep* **7**, 1–13 (2017).

36. Zhao, Y. *et al.* Searching for a 'hidden' prophage in a marine bacterium. *Appl Environ Microbiol* **76**, 589–595 (2010).

37. Giovannoni, S. J. SAR11 Bacteria: The Most Abundant Plankton in the Oceans. *Ann Rev Mar Sci* **9**, 231–255 (2017).

38. Chen, L.-X. *et al.* Wide Distribution of Phage That Infect Freshwater SAR11 Bacteria. *mSystems* **4**, 1–16 (2019).

39. Kruger, D. H. & Bickle, T. A. Bacteriophage survival: Multiple mechanisms for avoiding the deoxyribonucleic acid restriction systems of their hosts. *Microbiol Rev* **47**, 345–360 (1983).

40. Hampton, H. G., Watson, B. N. J. & Fineran, P. C. The arms race between bacteria and their phage foes. *Nature* **577**, 327–336 (2020).

41. Hatfull, G. F. Unintelligent Design: Mosaic Construction of Bacteriophage Genomes. in *Life in Our Phage World: A centennial field guide to the Earth's most diverse inhabitants* (eds. Rohwer, F. L., Youle, M., Maughan, H. & Hisakawa, N.) 5–47 (Wholon, San Diego, CA, 2014).

42. Mizuno, C. M., Rodriguez-Valera, F., Kimes, N. E. & Ghai, R. Expanding the Marine Virosphere Using Metagenomics. *PLoS Genet* **9**, e1003987 (2013).

43. Martinez-Hernandez, F. *et al.* Single-virus genomics reveals hidden cosmopolitan and abundant viruses. *Nat Commun* **8**, 15892 (2017).

44. Son, M. & Serwer, P. Role of Exonuclease in the Specificity of Bacteriophage T7 DNA Packaging. *Virology* **190**, 824–833 (1992).

45. Zhao, Y., Qin, F. & Zhang, R. Pelagiphages in the Podoviridae family integrate into host genomes. *Environ Microbiol* **21**, 1989–2001 (2019).

46. Buchholz, H. H. *et al.* Efficient dilution-to-extinction isolation of novel virus–host model systems for fastidious heterotrophic bacteria. *ISME Journal* **15**, 1585–1598 (2021).

47. Smyth, T. J. *et al.* Ocean net heat flux influences seasonal to interannual patterns of plankton abundance. *PLoS One* **9**, (2014).

48. Breitbart, M., Bonnain, C., Malki, K. & Sawaya, N. A. Phage puppet masters of the marine microbial realm. *Nat Microbiol* **3**, (2018).

49. Cornillon, P. & Stramma, L. The distribution of diurnal sea surface warming events in the western Sargasso Sea. *J Geophys Res* **90**, 11811 (1985).

50. Lomas, M. W., Bates, N. R., Buck, K. N. & Knap, A. H. *Oceanography of the Sargasso Sea: Overview of Scientific Studies*. *Sargasso Sea Alliance Science Report Series* www.sargassoalliance.org (2011).

51. Hart, S. P., Schreiber, S. J. & Levine, J. M. How variation between individuals affects species coexistence. *Ecol Lett* **19**, 825–838 (2016).
