# Supplementary material for: Long-read powered viral metagenomics in the oligotrophic Sargasso Sea
Source: Nat Commun. 2024 May 14;15:4089. doi: 10.1038/s41467-024-48300-6 (PMC11094077; doi:10.1038/s41467-024-48300-6)
Supplement: Supplementary file 3 — Description of Additional Supplementary Files [file 41467_2024_48300_MOESM3_ESM.pdf]

Description of Additional Supplementary Files

Supplementary Dataset 1. Two-sided, pairwise Adonis F-test comparisons (PERMANOVA) of Euclidean distance between centroids of Sargasso Sea viruses and Global Ocean Virome (GOV2)[1] sample groups.

Reference: 1. Gregory, A. C. et al. (2019). Marine DNA Viral Macro- and Microdiversity from Pole to Pole. Cell, 177(5), 1109-1123.e14.

Supplementary Dataset 2. SIMPER analysis showed that 674 Sargasso Sea viruses (BATS-B, all of which were captured using long-read data) were important in discriminating the Sargasso Sea viromes from other viral communities/zones. Four members from these relatively rare viruses (bootstrapped median (*n* =10,000) number of Global Ocean Virome (GOV2) samples in which observed: 10 (4.5- 17 95% CI)) recruited a large number of reads from ≥1 temperate-tropical mesopelagic (TT-MES) or Antarctic (ANT) site implying some degree of viral import.

Supplementary Dataset 3. Metagenome Assembled Genomes (MAGs) generated from Sargasso Sea samples, including MAG Taxonomy, completion, and contamination.

Supplementary Dataset 4. Accession numbers (total: 438) of published cyanophage, pelagiphage and T4 genomes which were clustered to produce viral population representatives before recruitment of short reads recovered from Sargasso Sea viral- and cellular fraction samples.
