# Supplementary figures and images for: Long-read powered viral metagenomics in the oligotrophic Sargasso Sea
Source: Nat Commun. 2024 May 14;15:4089. doi: 10.1038/s41467-024-48300-6 (PMC11094077; doi:10.1038/s41467-024-48300-6)

BATS-B

Isolates

TT-EPI

TT-MES

ANT

BATS

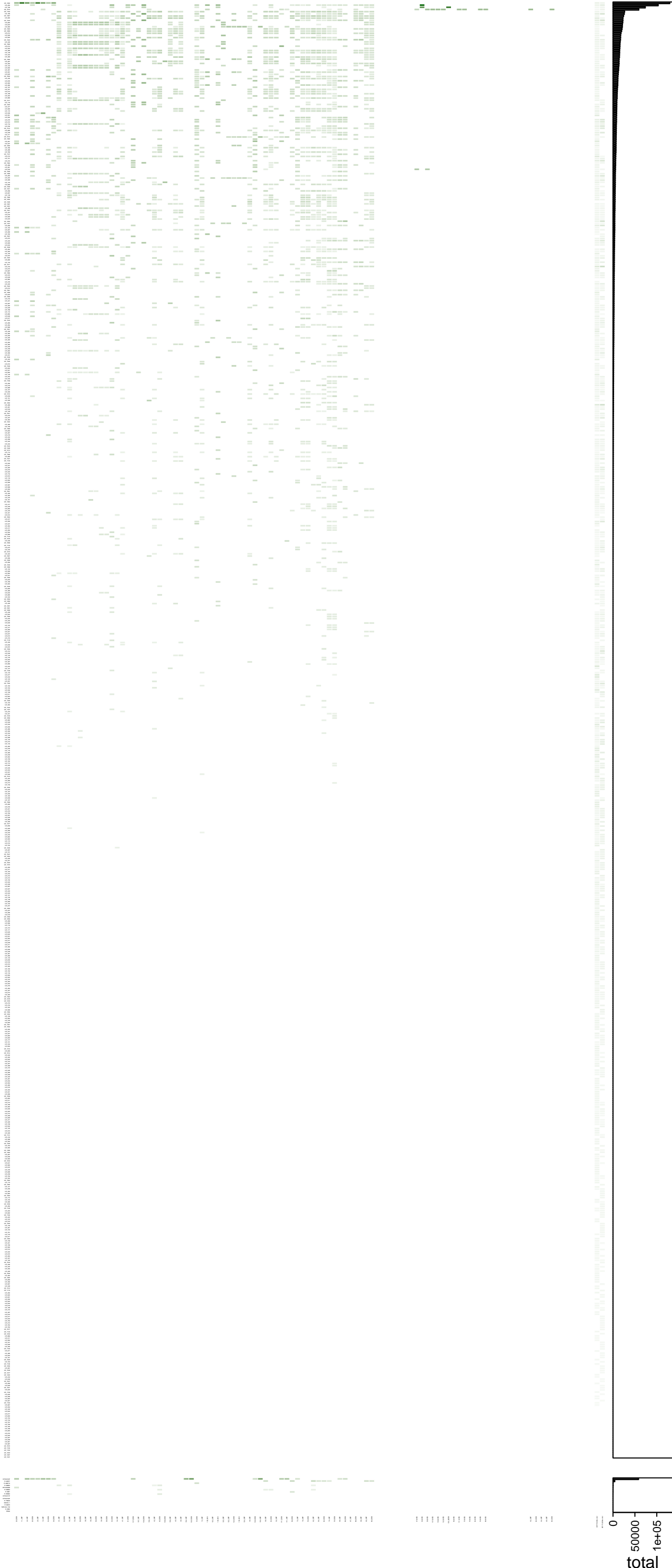

Supplement: Supplementary file 4 — Supplementary Datasets 1-4 [file 41467_2024_48300_MOESM4_ESM.zip › Supplementary_datasets_1-4/Supplementary_Dataset_2.pdf]
