# Supplementary material for: Long-read powered viral metagenomics in the oligotrophic Sargasso Sea
Source: Nat Commun. 2024 May 14;15:4089. doi: 10.1038/s41467-024-48300-6 (PMC11094077; doi:10.1038/s41467-024-48300-6)
Supplement: Supplementary file 5 — Reporting Summary [file 41467_2024_48300_MOESM5_ESM.pdf]

nature portfolio

Corresponding author(s): Joanna Warwick-Dugdale
Last updated by author(s): Apr 8, 2024

Reporting Summary

Nature Portfolio wishes to improve the reproducibility of the work that we publish. This form provides structure for consistency and transparency in reporting. For further information on Nature Portfolio policies, see our Editorial Policies and the Editorial Policy Checklist.

Statistics

For all statistical analyses, confirm that the following items are present in the figure legend, table legend, main text, or Methods section.

n/a	Confirmed	
☐	☒	The exact sample size (*n*) for each experimental group/condition, given as a discrete number and unit of measurement
☐	☒	A statement on whether measurements were taken from distinct samples or whether the same sample was measured repeatedly
☐	☒	The statistical test(s) used AND whether they are one- or two-sided<br>*Only common tests should be described solely by name; describe more complex techniques in the Methods section.*
☒	☐	A description of all covariates tested
☒	☐	A description of any assumptions or corrections, such as tests of normality and adjustment for multiple comparisons
☐	☒	A full description of the statistical parameters including central tendency (e.g. means) or other basic estimates (e.g. regression coefficient) AND variation (e.g. standard deviation) or associated estimates of uncertainty (e.g. confidence intervals)
☐	☒	For null hypothesis testing, the test statistic (e.g. *F*, *t*, *r*) with confidence intervals, effect sizes, degrees of freedom and *P* value noted<br>*Give P values as exact values whenever suitable.*
☒	☐	For Bayesian analysis, information on the choice of priors and Markov chain Monte Carlo settings
☒	☐	For hierarchical and complex designs, identification of the appropriate level for tests and full reporting of outcomes
☒	☐	Estimates of effect sizes (e.g. Cohen's *d*, Pearson's *r*), indicating how they were calculated

Our web collection on statistics for biologists contains articles on many of the points above.

Software and code

Policy information about availability of computer code

Data collection	Software pre-installed by manufacturers of the HiSeq 2500 ('HiSeq Analysis Software (HAS); Illumina Inc.) and rosette-CTD were used for data collection. Oxford Nanopore software 'MinKnow' was used to collect long-read data (version commiserate with December 2017).
Data analysis	Code for analyses and associated data used in the current study are available at:<br>https://github.com/BIOS-SCOPE/AE1712-viromes<br>https://doi.org/10.5281/zenodo.10940125118<br><br>QC Illumina data: cutadapt (https://github.com/marcelm/cutadapt/); (Martin, 2011); bbmap (https://jgi.doe.gov/data-and-tools/bbtools/)<br>Assembly Illumina data: metaSPAdes (v3.13.1; (Nurk et al., 2017)<br>Basecalling Nanopore data: Guppy v3.3.0 (ONT)<br>QC and demultiplexing Nanopore data: Porechop (v0.4; https://github.com/rrwick/Porechop); NanoFilt (De Coster et al., 2018)<br>Assembly and polishing Nanopore data: metaFlye (Kolmogorov et al., 2020); Minipolish (Vaser et al., 2017); Racon (Wick & Holt, 2019),Medaka (https://nanoporetech.github.io/medaka/)<br>Identification of putative viruses: VirSorter (v1.0.5; (Roux et al., 2015) (after augmenting its database with Xfam database of viral HMM profiles from (Guo et al., 2021))<br>Viral contig dereplication into viral populations; clustering with GOV2 data: ClusterGenomes (Roux et al., 2017) (https://github.com/simroux/ClusterGenomes)<br>Viral Population cluster member contig mapping to representative contigs: Minimap2; (Li, 2016)<br>Viral relative abundance calculation (via short-read recruitment) to viral contigs: Bowtie2 (Langmead & Salzberg, 2012); parsing and filtering: BamM (https://github.comEcogenomics/BamM) and CoverM (https://github.com/wwood/CoverM)

Total relative abundance of long-read derived and short-read derived viral populations, and differences between viral population lengths was tested and plotted with the packages tidyverse, cowplot, and scales, in R (https://www.R-project.org/).
MAGs: mapping of short reads to cellular contigs: CoverM (v0.2.0) (https://github.com/wwood/CoverM); binning: UniteM (v0.0.18) (UniteM; unpublished https://github.com/dparks1134/UniteM); DAS Tool (v1.1.1) (Sieber et al., 2018); MetaWRAP (v1.0.6) (Uritskiy et al., 2018); MetaBAT2 (Kang et al., 2019); GroopM2 (Imelfort et al., 2014); MaxBin2 (Wu et al., 2015); Evaluation (completeness, contamination, outliers): CheckM (v1.0.12) (Parks et al., 2015); RefineM (v0.0.24) (Parks et al., 2017); Taxonomical classification: GTDB-tk18 (Chaumeil et al., 2020)
Virus classification: Prodigal (v2.6.1; (Hyatt et al., 2010)
Viral Genera clustering: vConTACT2 (Bin Jang et al., 2019)
Attempted linkage of viral populations to hosts: prophage BLAST, tRNAscan-SE (v1.23) (Chen et al., 2019), and WIsH (v1.0) (Galiez et al., 2017)
Screening for marker genes: DRAM-v (Shaffer et al., 2020)
Assessment of gene completeness: CheckV v0.3.0 (Nayfach et al., 2020)
TerL genes alignment: MAFFT v7.017 (Katoh & Standley, 2016); trimming of aligned sequences: Trimal v1.4.rev15 (Capella-Gutiérrez, Silla-Martínez & Gabaldón, 2009) ; manual check for alignment overhangs: Geneious v10.2.6 (Kearse et al., 2012); appropriate substitution model determination: Model Finder (Kalyaanamoorthy et al., 2017); phylogenetic tree construction: IQ-tree (Nguyen et al., 2015); phylogeny visualisaiton: iTOL v5 (Letunic & Bork, 2016)
16S rRNA sequences trimmed, dereplicated, chimera checked, and ASV generating using the DADA2 package, version 1.2 (Callahan et al. 2016) in R (https://www.R-project.org/).
Taxonomy of 16S rRNA ASVs was assigned with the Silva database, version 123 (Quast et al. 2013).
Amplicon datasets were extracted with Phyloseq (McMurdie et al. 2013) and relative contributions were plotted using the ggplot package (Wickham 2009) in R (https://www.R-project.org/).
Community diversity: Vegan (Oksanen et al., 2020) (https://CRAN.R-project.org/package=vegan) and pracma (https://cran.r-project.org/web/packages/pracma/index.html) packages, with pairwise comparisons between groups package pairwiseAdonis (Arbizu, 2020) in R (https://www.R-project.org/).
Presence/absence of Sargasso Sea viral populations in GOV2 dataset and normalised levels of nitrogen and Phosphate were plotted using ggplot package (Wickham 2009) in R (https://www.R-project.org/). Global distribution of Sargasso Sea samples was visualised with the R (https://www.R-project.org/) packages Simple Features (Pebesma, 2018) and rnaturalearth (https://github.com/ropensci/rnaturalearth), and edited with vector editor Inkscape (Inkscape Project, 2024).
Global relative abundance of Sargasso Sea long-read viruses and isolates that infect SAR11 and Prochlorococcus plotted using packages tydyverse (Wickham et al. 2019), cowplot (Wilke, 2024), scales (Wickham et al. 2023), and ComplexHeatmap (Gu et al. 2016) in R (https://www.R-project.org/); addition of Ocean and sea regions via editing in Inkscape.

Code for microdiversity bootstrapping analysis from: 'https://raw.githubusercontent.com/btemperton/tempertonlab_utils/master/R/StatsUtilities.R'
Random subsampling of viromes: bbmap (https://sourceforge.net/projects/bbmap/).
Single nucleotide variants (SNVs) calling: metapop (Gregory et al., 2020)
Per-nucleotide coverage of the top 50 most abundant viruses (for HVR analysis): bedtools2 (https://github.com/arq5x/bedtools2)
Bootstrapping and permutation tests: data manipulation and plotting with packages Tidyverse (Wickham et al. 2019), cowplot (Wilke, 2024), Colorblindr(https://rdocumentation.org/packages/colorblindr/versions/0.1.0), Colorspace (Ihaka et al. 2016) and Scales (Wickham et al. 2023) in R (https://www.R-project.org/).
Investigation of functional encoding within candidate HVRs: tBLASTx
Fragmentation and recovery of long-read derived viral population representatives by short-read derived contigs against their relative abundances: RPKM values calculated CoverM, v0.2.0 (https://github.com/wwood/CoverM) plotting: packages ggplot (Wickham 2009), tidyverse (Wickham et al. 2019), cowplot (Wilke, 2024),colorspace (Ihaka et al. 2016) and ggrepel (Slowikowski, 2024), in R (https://www.R-project.org/).
Analyses to determine whether previously isolated viruses of cyanobacteria and SAR11 were represented in Sargasso Sea viromes (from this study) and cellular metagenomes (from this study and previous studies): cyanophage and pelagiphage isolate genomes (accession numbers: Supplementary Dataset 4) viral population representatives generated using ClusterGenomes (Roux et al. 2017); short reads were recruited using Bowtie2 (Langmead et al. 2012); bam files parsed in CoverM (https://github.com/wwood/CoverM); RPKM calculated as above. Data manipulation and plotting conducted using packages Tidyverse (Wickham et al. 2019), cowplot (Wilke, 2024) and colorspace (Ihaka et al. 2016) in R (https://www.R-project.org/).

REFERNCES: See paper.

For manuscripts utilizing custom algorithms or software that are central to the research but not yet described in published literature, software must be made available to editors and reviewers. We strongly encourage code deposition in a community repository (e.g. GitHub). See the Nature Portfolio guidelines for submitting code & software for further information.

Data

Policy information about availability of data

All manuscripts must include a data availability statement. This statement should provide the following information, where applicable:

- Accession codes, unique identifiers, or web links for publicly available datasets
- A description of any restrictions on data availability
- For clinical datasets or third party data, please ensure that the statement adheres to our policy

DATA AVAILABILITY
The sequencing data and assemblies generated in this study have been deposited in the National Center for Biotechnology Information (NCBI) database under the BioProject accession code PRJNA767318 [https://www.ncbi.nlm.nih.gov/bioproject/?term=PRJNA767318].
The amplicon raw SRA data used in this study are available in the NCBI database under the BioProject accession code PRJNA769790 [https://www.ncbi.nlm.nih.gov/bioproject/?term=PRJNA769790].
The following data generated in this study are provided in the Supplementary Information: PERMANOVA analysis results (Supplementary Dataset 1); 'BATS-B' (from SIMPER analysis) contig abundance (Supplementary Dataset 2); Metagenome Assembled Genomes (MAGs) (Supplementary Dataset 3); Accession numbers of

published cyanophage, pelagiphage and T4 genomes used in the production of Supplementary Figure 7 (Supplementary Dataset 4).
The following data generated in this study are provided in the Source Data file:
Alignments of Sargasso Sea long-read derived viral population representatives against short-read derived population members; used as input for the script calc_breakages.py, towards production of Supplementary Figures 2A and B (alignment_short_members_t0_long_reps.txt);
Areas of Sargasso Sea long-read derived viral population representatives not aligned to short-read population members; output of calc_breakages.py, towards production of Supplementary Figures 2A and B (LRR_breakages_2023_02_23.csv);
Relative abundance (RPKM) of Sargasso Sea long-read derived viral population representatives in short-read data; towards production of Supplementary Figures 2A and B (long_read_cluster_rep_rel_abundance_covminzero.txt);
Alignments of Sargasso Sea long-read derived viral population representatives against short-read contigs <1kb in length; used as input for the script calc_breakages.py, towards production of Supplementary Figure 2C (alignment_shortread_contigs_gr1kb_to_all_LRR-sorted-by-target-name.txt);
Areas of Sargasso Sea long-read derived viral population representatives not aligned to short-read contigs <1kb; Output of calc_breakages.py; towards production of Supplementary Figure 2C (breakages_all_LRR_contigs_gr_1kb.csv);
Relative abundance (RPKM) of Sargasso Sea long-read derived viral population representatives in short-read data; towards production of Supplementary Figure 2C (All_long_read_cluster_rep_RPKM.txt);
Abundance tables generated from mapping Sargasso Sea reads and GOV2 reads to Sargasso Sea population representative contigs, plus GOV2 metadata; used in production of Figures 3 and 5a. (folder figure5a_figure3);
Microdiversity values for Sargasso Sea contigs used to produce Supplementary Figures 9, 10, and 11 (sl_contig_microdiversity.tsv);
Viral population representative contig names, lengths and type; used for production of Figure 1 (length_2301.txt);
Rank and abundance values for short-read viral population representatives; used for production of Figure 1 and Figure 5 (‘rank_abundance_2301.csv);
Metadata (including labels) for GOV2 and Sargasso Sea samples; used for production of Figure 3 (BATS_GOV2.0_env.cvs);
Short-read coverage of GOV2 and Sargasso Sea viral populations; used for production of Figure 3 (GOV2.0_BATS_coverage);
Metadata (including labels) for Sargasso Sea samples; used for production of Figure 5 and Supplementary Figures 3, 4 and 5 (BATS_env.csv);
Short-read coverage of Sargasso Sea viral populations; used for production of Figure 5A (BATS_short_reads_2031_coverage_rm2v.csv);
Short-read coverage of Sargasso Sea viral populations; used for production of Supplementary Figure 3A (GOV2.0_BATS_coverage_S3A.csv);
Short-read coverage of Sargasso Sea viral populations; used for production of Supplementary Figure 3B (GOV2.0_BATS_coverage_S3B.csv)
Short-read coverage of Sargasso Sea viral populations from short-read sequencing; used for production of Supplementary Figure 4 (BATS_short_reads_1044_coverage.csv)

Code for analyses and associated data used in the current study are available at:
https://github.com/BIOS-SCOPE/AE1712-viromes
https://doi.org/10.5281/zenodo.10940125118

Research involving human participants, their data, or biological material

Policy information about studies with human participants or human data. See also policy information about sex, gender (identity/presentation), and sexual orientation and race, ethnicity and racism.

Reporting on sex and gender	This study does not involve human research participants; this type of information was not collected.
Reporting on race, ethnicity, or other socially relevant groupings	See above.
Population characteristics	See above.
Recruitment	See above.
Ethics oversight	See above.

Note that full information on the approval of the study protocol must also be provided in the manuscript.

Field-specific reporting

Please select the one below that is the best fit for your research. If you are not sure, read the appropriate sections before making your selection.

☐ Life sciences ☐ Behavioural & social sciences ☒ Ecological, evolutionary & environmental sciences

For a reference copy of the document with all sections, see nature.com/documents/nr-reporting-summary-flat.pdf

Ecological, evolutionary & environmental sciences study design

All studies must disclose on these points even when the disclosure is negative.

Study description	Metagenomic samples from pelagic microbial cellular communities and viral assemblages were collected from the Sargasso Sea (Bermuda Atlantic Time Series: BATS) via rosette-mounted Niskin bottles at 12-hour intervals coinciding with dusk and dawn (two factors), from depths of 80 m and 200 m (two factors), over a period of four consecutive days. Dusk and Dawn samples from consecutive days were treated as replicates (n=3) due to water-budget constraints. A continuous variable of time was not considered in the analyses. In summary, each combination of Dusk/Dawn vs depth had three replicate samples. Each sample comprised cellular and viral fractions (two factors). Cellular communities were recovered onto 0.22 µm pore Sterivex filters via filtration of 5 L of seawater per sample. Viral assemblages were obtained via sequential filtration of 20 L seawater per sample, followed by iron chloride

	flocculation (John et al., 2011), with modifications for prevention of DNA degradation and removal of PCR inhibitors (Warwick-Dugdale et al., 2019b). Short-read Illumina sequencing was performed on all cellular community DNA and viral assemblage DNA. In addition, long-read sequences were generated from viral metagenomic DNA collected from the depth of 80m (n=3) using the 'VirION' pipeline (Warwick-Dugdale et al., 2019b). Samples from 200m did not amplify sufficiently for preparation of long-read sequencing libraries.
Research sample	Sargasso Sea metagenomic microbial cellular populations and Sargasso Sea metagenomic viral populations, at depths of 80 m and 200 m, were sampled during summer stratification of the water column. Previous understanding of Sargasso Sea viral community structure is limited to seasonal patterns in abundances of viral-like particles (Parsons et al., 2012) and genomic analysis of isolated phages (Sullivan et al., 2005, 2010; Kelly et al., 2013; Zhao et al., 2013; Buchholz et al., 2021a). Here, we used metagenomics to characterise the viral communities at 80m (depth of maximum viral particle abundance) and 200m (mesopelagic; depth of recalcitrant carbon specialists 'SAR202') in both the cellular and viral fractions of the summer-stratified Sargasso Sea. Long read viral fraction sequencing was used to overcome assembly fragmentation due to microdiversity and to improve recovery of virally encoded HVRs to facilitate evaluation of their role in niche-adaptation. After dereplication of identified viral contigs, the global distribution of Sargasso Sea viruses was investigated using the Global Ocean Virus (GOV) 2.0 dataset (Gregory et al., 2019), which contains 145 viral metagenomic samples from five distinct global ecological zones, including the Arctic, Antarctic, bathypelagic, temperate and tropical epipelagic, and mesopelagic. Classification of Sargasso Sea viruses was attempted via genera-level clustering alongside RefSeq prokaryotic viral genomes (release 88) for reference to known isolates. To identify pelagiphages, terminase (TerL) genes were identified from published pelagiphage genomes, non-pelagiphages (from NCBI refseq, search term: 'marine terminase in viruses'), Pelagibacterales (identified via BLAST hits against terminase from known pelagiphages) and Sargasso Sea viral populations. To investigate the content of candidate hypervariable regions, functions encoded within HVRs were investigated using a tBLASTx search against the NCBI NR database.
Sampling strategy	Metagenomic samples were collected aboard the RV Atlantic Explorer at the Bermuda Atlantic Time Series (BATS; http://bats.bios.edu/) station (31°40'N, 64°10'W) via rosette-mounted Niskin bottles during dusk (~19:00 local time) and dawn (~06:00 local time), from depths of 80 m and 200 m, over a period of four consecutive days from the 8th-11th July 2017. Cellular communities (n = 12) were obtained from 5 L of seawater per sample transferred immediately to clean polycarbonate bottles; the cellular fraction was recovered onto 0.22 µm pore Sterivex filters via positive pressure filtration. Viral assemblages (n = 12) were obtained via sequential filtration of 20 L seawater per sample, followed by iron chloride flocculation (John et al., 2011); Briefly, peristaltic pumps and 142 mm rigs were used to remove the cellular fraction via sequential filtering through glass fibre (GF/D: pore size 2.7 µm) then polyethersulfone (pore size 0.22 µm) filters, before flocculation and precipitation of viruses via iron chloride. Iron-bound viral particle flocculate was recovered onto 1.0 µm polycarbonate filters (within 4 hours of collection). Sample sizes were chosen to provide sufficient DNA for sequencing of microbial and viral communities, and agree with previously published methods (e.g. Roux at al, 2016). Sequencing depth also effects the sample size, so samples from this study and Global Ocean samples were subsampled to the same depth for analyses of abundance.
Data collection	Environmental samples and associated environmental data (e.g. sampling depth) was collected via CTD probes attached to a Niskin rosette by members of the 'BIOS-SCOPE' project, including Joanna Warwick-Dugdale, during cruise sampling procedure. The Sequencing data was collected via sequencing software (e.g. Nanopore: 'MinKnow'), and was curated as described in the 'Methods' and 'Supplemental Methods' of the manuscript.
Timing and spatial scale	Sampling took place over a period of four consecutive days during the BIOS-SCOPE Summer Cruise of 2017; Data collection start: 8th-July 2017; data collection finish: 11th July 2017. Timing of data collection: dusk (~19:00 local time) and dawn (~06:00 local time), chosen to investigate possible signal of diurnal cycles (none recorded; data not shown). Spatial scale: samples collected on the Bermuda Atlantic Time Series (BATS; http://bats.bios.edu/) station (31°40'N, 64°10'W), at depths of 80 m and 200 m.
Data exclusions	One viral sample (collected July 8th from 200m) was excluded from further investigation due to a lack of viral sequence detection, the result of very low sequencing depth (29.6 Mbp, 100 times smaller than that of other samples; exclusion criteria not pre-estabilished). Long-reads were excluded from analysis to avoid potential artefacts associated with the sequencing technology in the following: clustering and number of viruses based on sample fraction type (i.e. cellular; free-particulate).
Reproducibility	No attempts have been made to repeat the experiment at this time.
Randomization	Samples were not allocated into groups beyond the environmental parameters of their collection (e.g. depth; sample fraction).
Blinding	Blinding was not relevant to this study as all samples were processed uniformly, according to sample type, and the sequencing data acquired is automatically collected via sequencing software. Software parameters and data curation were applied uniformly for the different data types (e.g. all short-read data underwent the same QC pipeline; all viral data was passed through VirSorter for identification of putative viral contigs).

Did the study involve field work? ☒ Yes ☐ No

Field work, collection and transport

Field conditions	Study conditions were typical for summer on the Sargasso Sea at the BATS station: zero precipitation; seawater surface temperature: >25 degrees Celsius. More information is available from http://bats.bios.edu
Location	Bermuda Atlantic Time Series (BATS; http://bats.bios.edu/) station (31°40'N, 64°10'W); sampling at depths of 80 m and 200 m.
Access & import/export	The samples were collected during the Summer 2017 BIOS-SCOPE cruise. The BIOS 'Collection, Export and Experimental Policies (CEEP)' sates: "BIOS is committed to preserving Bermuda's natural environment. In consultation with the Bermuda Government, BIOS has established detailed protocols for the collection, export and experimental manipulation of materials (plants, animals, geological).

	All materials collected must first be approved by the BIOS CEEP Committee (unless covered by the Limited Impact Research policy) and then the Bermuda Government (unless covered by the Limited Impact Research policy)." Export permit via BIOS internal request, BIOS, 17 Biological Station, St George's, Bermuda GE01.
Disturbance	Collection of pelagic samples via CTD-Niskin Rosette likely to cause minimal disturbance; boat engine noise and presence possible disturbance to local macro-megafauna (none observed on Station). Disturbance minimised by efficient sampling schedule and prompt and return to Bermuda.

Reporting for specific materials, systems and methods

We require information from authors about some types of materials, experimental systems and methods used in many studies. Here, indicate whether each material, system or method listed is relevant to your study. If you are not sure if a list item applies to your research, read the appropriate section before selecting a response.

Materials & experimental systems

n/a	Involved in the study
☒	☐ Antibodies
☒	☐ Eukaryotic cell lines
☒	☐ Palaeontology and archaeology
☒	☐ Animals and other organisms
☒	☐ Clinical data
☒	☐ Dual use research of concern
☒	☐ Plants

Methods

n/a	Involved in the study
☒	☐ ChIP-seq
☒	☐ Flow cytometry
☒	☐ MRI-based neuroimaging

Plants

Seed stocks	The study did not involve seed stocks or other plant material.
Novel plant genotypes	The study did not involve novel plant genotypes
Authentication	This study did not involve novel seed stocks or plant genotypes.
