# Supplementary material for: Long-read powered viral metagenomics in the oligotrophic Sargasso Sea
Source: Nat Commun. 2024 May 14;15:4089. doi: 10.1038/s41467-024-48300-6 (PMC11094077; doi:10.1038/s41467-024-48300-6)
Supplement: Supplementary file 6 — Source Data file [file 41467_2024_48300_MOESM6_ESM.zip › source_data/source_data_contents.docx]

**Long-read powered viral metagenomics in the Oligotrophic Sargasso Sea: Source Data file Contents:**

| **File Name** | **Description** |
| --- | --- |
| alignment_short_members_t0_long_reps.txt | Alignments of Sargasso Sea long-read derived viral population representatives against short-read derived population members; used as input for the script calc_breakages.py, towards production of Supplementary Figures 2A and B |
| LRR_breakages_2023_02_23.csv | Areas of Sargasso Sea long-read derived viral population representatives not aligned to short-read population members; output of calc_breakages.py, towards production of Supplementary Figures 2A and B |
| long_read_cluster_rep_rel_abundance_covminzero.txt | Relative abundance (RPKM) of Sargasso Sea long-read derived viral population representatives in short-read data; towards production of Supplementary Figures 2A and B |
| alignment_shortread_contigs_gr1kb_to_all_LRR-sorted-by-target-name.txt | Alignments of Sargasso Sea long-read derived viral population representatives against short-read contigs <1kb in length; used as input for the script calc_breakages.py, towards production of Supplementary Figure 2C |
| breakages_all_LRR_contigs_gr_1kb.csv | Areas of Sargasso Sea long-read derived viral population representatives not aligned to short-read contigs <1kb; Output of calc_breakages.py; towards production of Supplementary Figure 2C |
| All_long_read_cluster_rep_RPKM.txt | Relative abundance (RPKM) of Sargasso Sea long-read derived viral population representatives in short-read data; towards production of Supplementary Figure 2C |
| folder figure5a_figure3 | Abundance tables generated from mapping Sargasso Sea reads and GOV2 reads to Sargasso Sea population representative contigs, plus GOV2 metadata; used in production of Figures 3 and 5a |
| sl_contig_microdiversity.tsv | Microdiversity values for Sargasso Sea contigs used to produce Supplementary Figures 9, 10, and 11 |
| length_2301.txt | Viral population representative contig names, lengths and type; used for production of Figure 1 |
| rank_abundance_2301.csv | Rank and abundance values for short-read viral population representatives; used for production of Figure 1 and Figure 5 |
| BATS_GOV2.0_env.cvs | Metadata (including labels) for GOV2 and Sargasso Sea samples; used for production of Figure 3 |
| GOV2.0_BATS_coverage | Short-read coverage of GOV2 and Sargasso Sea viral populations; used for production of Figure 3 |
| BATS_env.csv | Metadata (including labels) for Sargasso Sea samples; used for production of Figure 5 and Supplementary Figures 3, 4 and 5 |
| BATS_short_reads_2031_coverage_rm2v.csv | Short-read coverage of Sargasso Sea viral populations; used for production of Figure 5A |
| GOV2.0_BATS_coverage_S3A.csv | Short-read coverage of Sargasso Sea viral populations; used for production of Supplementary Figure 3A |
| GOV2.0_BATS_coverage_S3B.csv | Short-read coverage of Sargasso Sea viral populations; used for production of Supplementary Figure 3B |
| BATS_short_reads_1044_coverage.csv | Short-read coverage of Sargasso Sea viral populations from short-read sequencing; used for production of Supplementary Figure 4 |
